# Low birth weight and its associated risk factors: Health facility-based case-control study

**DOI:** 10.1371/journal.pone.0234907

**Published:** 2020-06-22

**Authors:** Anil K. C., Prem Lal Basel, Sarswoti Singh

**Affiliations:** 1 Health Foundation Nepal, Patan, Nepal; 2 Department of Community Medicine and Public Health, Tribhuvan University Teaching Hospital, Kathmandu, Nepal; Tribhuvan University Institute of Medicine, NEPAL

## Abstract

**Background:**

Low birth weight is a preventable public health problem. It is an important determinant of child survival and development, as well as long-term consequences like the onset of non-communicable disease in the life course. A large number of mortality and morbidity can be prevented by addressing the factors associated with low birth weight. The main objective of this study was to identify associated risk factors of low birth weight.

**Methodology:**

A health facility-based unmatched case-control study was carried out from July 2018 to March 2019 among the mothers who delivered in health facilities of Dang district of Nepal from 17^th^ August to 16^th^ November 2018. The total sample size for the study was 369; 123 cases and 246 controls. Cases and controls were randomly selected independent of the exposure status in the ratio of 1:2. Information regarding exposure status was assessed through interviews and medical records. Mothers who delivered outside Dang districts were excluded from the study. Ethical clearance was obtained from the Institutional Review Committee (IRC) of the Institute of Medicine, Tribhuvan University and written consent was taken from each participant after explaining the objectives of the study.

**Results:**

Multivariate logistic regression found that having the kitchen in the same living house (AOR 2.7, CI: 1.5–4.8), iron intake less than 180 tablets (AOR 3.2, CI: 1.7–5.7), maternal weight gain during second and third trimester less than 6.53 kg (AOR 2.6, CI: 1.5–4.7), co-morbidity during pregnancy (AOR 2.4, CI: 1.3–4.5), preterm birth (AOR 2.9, CI: 1.4–6.1) were the risk factors associated with low birth weight.

**Conclusion:**

Having the kitchen in the same living house, iron intake less than 180 tablets during pregnancy, maternal weight gain less than 6.53 kg during the second and third trimester, co-morbidity during pregnancy and preterm birth were the risk factors associated with low birth weight.

## Introduction

World Health Organization defines low birth weight (LBW) as the birth weight less than 2500 grams irrespective of gestational age [1]. LBW is a valuable public health indicator of maternal health, nutrition, healthcare delivery, and poverty as LBW babies are at a higher risk of death and illness shortly after birth and non-communicable disease in the life course [2]. LBW infants are 20 times more likely to develop complications and die in comparison to normal weight babies [3]. LBW babies are in the potential risk of cognitive deficits, motor delays, cerebral palsy, and other behavior and psychological problem [4–8]. The household cost, as well as health system costs, could be saved by reducing the burden of LBW [9]. The pathophysiology of low birth weight is unclear, whereas intrauterine growth retardation (IUGR) and preterm birth considered as the cause of LBW. IUGR is the outcome of insufficient uterine–placental perfusion and fetal nutrition affecting the overall anthropometric parameter of the fetus. IUGR newborn has typical features of malnutrition. Extra-uterine infection, trauma, illness, IUGR, fetal infection, and anomalies are the contributing factors for preterm birth, resulting in growth retardation which ultimately results in LBW [3, 10, 11]. LBW is considered a significant public health problem as it is estimated that 15% to 20% of all birth worldwide are LBW. The prevalence of LBW varies across regions with the highest 28% in South Asia and the lowest 6% in East Asia and the Pacific region [12]. The prevalence of LBW in Nepal ranges from 12% to 21.6%, [13–15]. A few descriptive and hospital-based case-control studies have been done in Nepal [14–19]. These descriptive and hospital-based studies could not represent the risk factors of LBW at the community level as these studies had taken participants from hospitals only. Hence this study aims to identify the associated risk factors of LBW at the community level by including the participants from the community level health facilities.

## Methodology

An unmatched case-control study was used. This study was conducted in Dang district of Nepal. This study was approved by the Institutional Review Committee (IRC) of Institute of Medicine (IOM) Tribhuvan University on August 19, 2018. The study population was mothers, who delivered their babies in the governmental health institutions (28 birthing centers and 3 hospitals) of Dang from 17^th^ August to 16^th^ November 2018. The study population was divided into case and control as per the following definition.

### Case

Mother delivering singleton live-born baby with birth weight less than 2500 grams without any congenital anomalies and were originally from Dang district.

### Control

Mother delivering singleton live-born baby with birth weight more or equal to 2500 grams without any congenital anomalies and were from the same Dang district.

The sample size was calculated using EpiInfo software version 7. This was calculated by taking power at 80%, confidence level as 95%, the percentage of control exposed as 65.40, the odds ratio of 2.06 from the maternal weight against LBW [20], and the ratio of case to control was 1:2. The total sample size was 369 with 123 cases and 246 controls. The eligible numbers of participants were enlisted from the maternal and neonate health register of 28 birthing centers and 3 hospitals of Dang District. One hundred and twenty-three cases were selected from the list of 224 cases and 246 controls from the list of 777 controls randomly independent of exposure status by generating random numbers. The 123 cases and 246 controls with the highest random number were visited with the help of FCHV, local leaders, teachers for the data collection. Face to face interview was done with the participants for the collection of data using a semi-structured questionnaire. Information regarding weight gain, age, ANC visit, birth weight, comorbidity, gestational age etc was taken through reviewing Antenatal Care (ANC) card and Maternal and newborn register to avoid possible recall bias. The tool was adapted from the previous studies done in Nepal [16–18, 20, 21]. The tool was translated into the Nepali language and pretested in Dhulikhel municipality; of Kavrepalanchok district among 10 percent of sample size i.e. 12 cases and 24 controls.

Data entry was done in Epi data Version 3.1 following coding. Data analysis was done using SPSS software version 21. Bivariate associations between independent variables and low birth weight were tested through the Chi-square test and the association was analyzed by calculating crude odds ratios (OR) at 95% confidence interval through binary logistic regression. Multivariate logistic regression was examined for the relationship between independent variables and low birth weight to address the confounding effect. Hosmer and Lemeshow test was used to test the goodness-of-fit for regression models. The test statistic was 0.69 (p > 0.05) that showed that the model adequately fit the data.

Ethical clearance was obtained from the Institutional Review Committee (IRC) of the Institute of Medicine, Tribhuvan University. Permission was taken from the District Public Health Office (DPHO) Dang and respective health facilities. Written consent was taken from each participant after explaining the objectives of the study. After the interview, the mothers were informed about the importance of growth monitoring, exclusive breastfeeding, immunization, and appropriate time of weaning.

## Results

Table 1 depicts that the mean age of the participants was 23 years (SD 4.4 years). Getting support from their husbands in day to day activities during pregnancy was quite common. The major (66.4%) fuel used during cooking was firewood and kerosene. The majority (53.1%) of the household did not have a separate kitchen. Majority (55.3%) of participants' family members did not smoke any form of cigarette. A small portion (2.2%) of participants had the habit of smoking cigarettes during pregnancy. Two-third (67%) of participants had their meal thrice a day and 64.2% had included additional food groups in their meal at the time of pregnancy. Majority (78%) of participants had attended ANC visit as per the protocol of the government of Nepal. Similarly, 73% of participants had taken 180 or more iron tablets during pregnancy. More than half of the participants had gained weight less than 6.53 kg during the second and third trimester. Fifty-four percentages of participants had one child. Majority (59.3% among cases and 86.2% among controls) of the research participants did not face any health problems (co-morbidities) during their pregnancy and 14.6% of the babies were born before 37 weeks of gestation.

**Table 1:** Distribution of participants according to socio-demographic, maternal factors and co-morbidity during pregnancy.

Variables	Case (123)	Control (246)	Total (369)
	n (%)	n (%)	
Age of mother			
<20 Years	35 (28.5%)	55 (22.4%)	90 (24.4%)
20–30 Years	79 (64.2%)	178 (72.4%)	257 (69.6%)
>30 years	9 (7.3%)	13 (5.3%)	22 (6.0%)
Support from husband in day to day activities			
Yes	102 (82.9%)	222 (90.2%)	324 (87.8%)
No	21 (17.1%)	24 (9.8%)	45 (12.2%)
Cooking material			
Fire wood and Kerosene	87 (70.7%)	158 (64.2%)	245 (66.4%)
LPG and Bio Gas	36 (29.3%)	88 (35.8%)	124 (33.6%)
Location of kitchen			
Same house	86 (69.9%)	110 (44.7%)	196 (53.1%)
Separate house and outside	37 (30.1%)	136 (55.3%)	173 (46.9%)
Smoking by family member			
Yes	65 (52.8%)	100 (40.7%)	165 (44.7%)
No	58 (47.2%)	146 (59.3%)	204 (55.3%)
Smoking habit			
Yes	6 (4.9%)	2 (.8%)	8 (2.2%)
No	117 (95.1%)	244 (99.2%)	361 (97.8%)
Food frequency per day			
Twice	30 (24.4%)	44 (17.9%)	74 (20.1%)
Thrice	81 (65.9%)	166 (67.5%)	247 (67%)
More	12 (9.6%)	36 (14.6%)	48 (13.0%)
Type of food use			
As usual	56 (45.5%)	76 (30.9%)	132 (35.8%)
Addition food (Any group)	67 (54.5%)	170 (69.1%)	237 (64.2%)
ANC visit as per protocol			
Yes	80 (65.0%)	208 (84.6%)	288 (78.0%)
No	43 (35.0%)	38 (15.4%)	81 (22.0%)
Number of iron tablets used			
<180	60 (48.8%)	40 (16.3%)	100 (27.1%)
180 and more	63 (51.1%)	206 (83.7%)	269 (72.9%)
Weight gain between second and third trimester			
<6.53 kilogram (kg)	91 (74.0%)	120 (48.8%)	211 (57.2%)
6.53 kg and above	32 (26.0%)	126 (51.2%)	158 (42.8%)
Number of children			
1 (Primiparity)	79 (64.2%)	121 (49.2%)	200 (54.2%)
2	32 (26.0%)	93 (37.8%)	125 (33.9%)
3	8 (6.5%)	26 (10.6%)	34 (9.2%)
4 and above	4 (3.3%)	6 (2.4%)	10 (2.7%)
Health problem			
Yes	50 (40.7%)	34 (13.8%)	84 (22.8%)
No	73 (59.3%)	212 (86.2%)	285 (77.2%)
Preterm birth			
Yes	33 (26.8%)	21 (8.5%)	54 (14.6%)
No	90 (73.2%)	225 (91.5%)	315 (85.4%)

Table 2 shows the bivariate and multivariate analysis of dependent and independent variables. In bivariate analysis; support from husband during pregnancy, use of firewood and kerosene during cooking, having kitchen in the same living house, cigarette smoking by family members, cigarette smoking by mother during pregnancy, use of additional food groups in their diet during pregnancy, four ANC visit as per protocol of Government of Nepal, iron tablets intake less than 180 tablets during pregnancy, weight gain during pregnancy less than 6.53 kg in-between second and third trimester, mother delivering her first baby, health problem during pregnancy and preterm baby were associated with low birth weight.

**Table 2:** Bivariate and Multivariate analysis of independent variables against low birth weight.

Variables	p-value	Crude OR	CI (95%)	Adjusted OR	CI (95%)
Age of mother					
<20 Years	0.4	1.4	0.9 to 2.4	0.8	0.4 to 1.6
>30 years		1.6	0.6 to 3.8	1.5	0.4 to 5.3
20–30 Years		Ref		Ref	
Support from husband in day to day activities					
No	0.04	1.9	1.0 to 3.6	1.5	0.7 to 3.3
Yes		Ref		Ref	
Cooking material					
Fire wood and Kerosene	0.2	1.4	0.8 to 2.2	1.4	0.7 to 2.6
LPG and Bio Gas		Ref		Ref	
Location of kitchen (Proxy of indoor air pollution)					
Same house	<0.0001	2.9	1.8 to 4.6	2.7	1.5 to 4.8
Separate house and outside		Ref		Ref	
Smoking by family member					
Yes	0.026	1.6	1.1 to 2.5	1.4	0.8 to 2.5
No		Ref		Ref	
Smoking habit					
Yes	0.032	6.3	1.2 to 31.5	5.3	0.7 to 42.7
No		Ref		Ref	
Food frequency per day					
Twice	0.19	2	0.9 to 4.6	1.3	0.5 to 3.6
Thrice		1.5	0.7 to 3	1.5	0.7 to 3.5
More		Ref		Ref	
Type of food use					
As usual	0.006	1.9	1.2 to 2.9	0.9	0.5 to 1.6
Addition food (Any group)		Ref		Ref	
ANC visit as per the protocol of Nepal Government					
No	<0.0001	2.9	1.8 to 4.9	1.7	0.9 to 3.2
Yes		Ref		Ref	
Number of iron tablets used					
<180	<0.0001	4.8	3.0 to 7.9	3.2	1.7 to 5.7
180 and more		Ref		Ref	
Weight gain					
Less than 6.53 kg	< 0.0001	3	1.9 to 4.8	2.6	1.5 to 4.7
6.53 kg and above		Ref		Ref	
Number of children					
1 (Primiparity)	0.03	1.9	1.2 to 3.1	1.8	0.9 to 3.6
3		0.9	0.4 to 2.2	0.4	0.1 to 1.2
4 and above		1.9	0.5 to 7.3	0.6	0.1 to 3.8
2		Ref		Ref	
Health problem (Co-morbidity)					
Yes	<0.0001	4.3	2.6 to 7.1	2.4	1.3 to 4.5
No		Ref		Ref	
Preterm birth					
Yes	<0.0001	3.9	2.2 to 7.2	2.9	1.4 to 6.1
No		Ref		Ref	

In multivariate analysis, having kitchen in the same living house (AOR 2.7, CI: 1.5–4.8), Iron intake less than 180 tablets (AOR 3.2, CI: 1.7–5.7), maternal weight gain less than 6.53 kg during second and third trimester (AOR 2.6, CI: 1.5–4.7), co-morbidities during pregnancy (AOR 2.4, CI: 1.3–4.5) and preterm birth (AOR 2.9, CI: 1.4–6.1) were significantly associated at 95% confidence interval with the low birth weight. Similarly, age of mother, support from husband during pregnancy, use of firewood and kerosene during cooking, smoking habit of the mother, smoking by family member, food frequency less than three per day, use of any additional food group during pregnancy, four ANC visit as per protocol, first children was not associated with LBW in this study.

## Discussion

This study analyzed the socio-demographic factors, maternal factors, and co-morbidities during recent pregnancy against low birth weight during delivery.

The maternal age is considered as a key factor for the healthy outcome of pregnancy. This study revealed no statistical association between maternal age and low birth weight which contradicts with the study done in Nepal, that shows a higher risk of delivering low birth weight babies by mother age less than 20 and more than 30 years [16, 17, 18]. Smoking during pregnancy had a negative effect on the growth and development of the fetus because of chemical substances present in it. Nicotine present in the cigarette cause vasoconstriction resulting in the low oxygen flow to the fetus and Carbon-monoxide forms carboxyhemoglobin which inhibits the oxygen release to fetal tissues [22] In bivariate analysis mother habit of smoking had a higher risk of low birth weight in reference to the mother who did not smoke a cigarette (OR 6.3, 95% CI: 1.2–31.5). This finding is consistent with the findings of similar studies done in Bangladesh and Turkey [23, 24]. Though there was a risk, however, there was no significant association between smoking and low birth weight in multivariate analysis. This could be explained probably due to the small number of smokers in the study population. Moreover, it can also be explained by the social desirability bias, induced due to social stigma.

This study identified the location of the kitchen in the living house, iron intake less than 180 tablets, weight gain less than 6.53 kg during the second and third trimester, comorbidity during pregnancy, and preterm birth as the risk factors for low birth weight. The finding reveals that the cooking fuels namely firewood and kerosene use had a risk for LBW with reference to LPG and Biogas however, it was not statistically significant. This finding contradicts to the find of the study done by Kadam YR et al. and Washam C [25, 26], however, this study revealed that having a kitchen in the same living house (proxy of indoor air pollution) had 2.5 times higher risk of delivering low birth weight which may be due to, living in the same house had higher risk and duration of exposure to the pollutants like PM_2.5_, PM_10_, NO_2_, SO_2_, CO caused by burning of fuels, leading to the impaired supply of oxygen, nutrition to the fetus resulting in the negative impact on the growth and development of fetus [27, 28]. The amount of exposure was not measured quantitatively in this study.

This study showed that total iron tablet intake during pregnancy was associated with the birth weight of the child. Mothers who took less than 180 tablets of iron during their pregnancy were three times more likely to deliver low birth weight babies with reference to mothers who took iron equal to or more than 180 tablets during their pregnancy period (AOR 3.2, CI: 1.7–5.8). This finding is similar to the studies conducted in Nepal [16, 29]. Low iron tablets intake causes the poor delivery of iron to the fetus thereby impair in proper hormonal and neuronal regulation of pregnancy and poor oxygenation to the fetus leading to the poor growth and development of the fetus [30]. However, the iron intake through diet during pregnancy was not measured in both cases and control.

The minimum standard weight gain during the second and third trimester is set as 6.53 kg [31]. Women who gained weight less than 6.53 kg during the second and third trimester had 3 times higher risk of delivering low birth weight baby with reference to women whose weight gain was 6.53 kg or above (AOR 2.8, CI: 1.6–5.0). This finding is similar to the study done in Bangladesh [32] and Mozambique [33]. The weight gain during pregnancy is impaired due to ill health, poor sanitation, and inadequate balance diet which at the end hamper the proper growth and development of the baby.

Women who had at least one health problem during their pregnancy were at higher risk of delivering low birth weight in comparison to women without any health problem (AOR 2.6, CI: 1.4–4.8). This finding is consistent with the study done in Nepal [17, 20]. Likewise, this study suggests that mother delivering baby before completion of 37 weeks of gestation had higher risk of delivering low birth weight than the mothers who deliver the term baby (AOR 2.6, CI: 1.2–5.5) which is in line with the study done in Nepal [17], Ethiopia [34] and Kenya [35]. Biologically it can be explained that preterm birth was less likely to get sufficient time for maturity, growth, and nutrient intake which therefore can lead to low birth weight [36].

In this study, the researcher has retrieved maternal information namely gestational weight, iron tablets intake, gestational age, co-morbidity, frequency of ANC visits, and birth weight of a baby from ANC card and maternity register to limit the recall bias. The selection of cases and controls were based on the records of maternal and neonatal register therefore, it is less likely that this study has misclassification biases both in the exposure and case-control categories. Controls were selected randomly independent of the exposure status and as there was no non-response in both the group, it is less likely that this study would suffer from selection biases. However, the study had some limitations. The findings might be influenced by social desirability bias. The findings could not be generalized as the study was confined in the health institutions of one district. The details of comorbidity during pregnancy, the micro-nutritional status of the mother, and the quality of ANC visit were not evaluated which may affect the outcome of this study.

## Conclusion and recommendation

This study concluded that the having the kitchen in the same living house (proxy of indoor air pollution), iron intake less than 180 tablets during pregnancy, weight gain less than 6.35 kg during the second and third trimester, co-morbidity during pregnancy, and preterm delivery were found to be associated risk factors of low birth weight. Thus, identified risk factors can be efficiently prevented through small doable actions that a family can apply and the mother can easily carry out. Maternal health programs can be directed towards motivating and tracking pregnant mothers for complete iron tablets intake during her pregnancy period. Intake of balance diet as per the protocol of the Government of Nepal for healthy growth and development of the child inside the uterus is of paramount importance. Family should help the mother for adequate rest, nutrition and healthy behavior to prevent risk factors identified in this study.

## Supporting information

S1 Data (SAV)
