# Supplementary material for: Low birth weight and its associated risk factors: Health facility-based case-control study
Source: PLoS One. 2020 Jun 22;15(6):e0234907. doi: 10.1371/journal.pone.0234907 (PMC7307746; doi:10.1371/journal.pone.0234907)
Supplement: Attachment — Submitted filename: PONE-D-19-31435_R1_reviewer.pdf [file pone.0234907.s006.pdf]

PLOS ONE

Low birth weight and its associated risk factors: health facility-based case-control study

--Manuscript Draft--

**Manuscript Number:**	PONE-D-19-31435R1
**Article Type:**	Research Article
**Full Title:**	Low birth weight and its associated risk factors: health facility-based case-control study
**Short Title:**	Low birth weight and its associated risk factors
**Corresponding Author:**	Anil K C<br>Health Foundation Nepal<br>Lalitpur, NEPAL
**Keywords:**	Low birth weight; Maternal Nutrition; Risk factors
**Abstract:**	Background: Low birth weight is a preventable public health problem. It is an important determinant of child survival and development, as well as long-term consequences like the onset of non-communicable disease in the life course. A large number of mortality and morbidity can be prevented by addressing the factors associated with low birth weight. The main objective of this study was to identify associated risk factors of low birth weight.<br>Methodology: A health facility-based unmatched case-control study was carried out from July 2018 to March 2019 among the mothers who delivered in health facilities of Dang district of Nepal from 17th August to 16th November 2018. The total sample size for the study was 369; 123 cases and 246 controls. Cases and controls were randomly selected independent of the exposure status in the ratio of 1:2. Information regarding exposure status was assessed through interviews and medical records. Mothers who delivered outside Dang districts were excluded from the study. Ethical clearance was obtained from the Institutional Review Committee (IRC) of the Institute of Medicine, Tribhuvan University and written consent was taken from each participant after explaining the objectives of the study.<br>Results: Multivariate logistic regression found that having the kitchen in the same living house (AOR 2.7, CI: 1.5-4.8), iron intake less than 180 tablets (AOR 3.2, CI: 1.7-5.7), maternal weight gain during second and third trimester less than 6.53 kg (AOR 2.6, CI: 1.5-4.7), co-morbidity during pregnancy (AOR 2.4, CI: 1.3-4.5), preterm birth (AOR 2.9, CI: 1.4-6.1) were the risk factors associated with low birth weight.<br>Conclusion: Having the kitchen in the same living house, iron intake less than 180 tablets during pregnancy, maternal weight gain less than 6.53 kg during the second and third trimester, co-morbidity during pregnancy and preterm birth were the risk factors associated with low birth weight.
**Order of Authors:**	Anil K C
	Prem Lal Basel
	Sarswoti Singh
**Response to Reviewers:**	Reviewer #1:<br>1. Introduction part needs more extensive literature review and rewrite your lines. eg- 44-45<br>Revised: LBW is a valuable public health indicator of maternal health, nutrition, healthcare delivery, and poverty as LBW babies are at a higher risk of death and illness shortly after birth and non-communicable disease in the life course.<br>Added: The household cost, as well as health system costs, could be saved by reducing the burden of LBW.<br>Added: IUGR is the outcome of insufficient uterine–placental perfusion and fetal nutrition affecting the overall anthropometric parameter of the fetus. IUGR newborn has typical features of malnutrition. Extra-uterine infection, trauma, illness, IUGR, fetal infection, and anomalies are the contributing factors for preterm birth, resulting in growth retardation which ultimately results in LBW.<br>2. Identify the risk factors of LBW from literature, and see the factors associated in your context. If you are trying to establish the association in your local settings, then it should fulfill the various criteria, it cannot be just a random association<br>Yes, variables were selected based on studies done in Nepali and outside in a similar

Powered by Editorial Manager® and ProduXion Manager® from Aries Systems Corporation

setting and consultation with experts and try to identify the possible risk factors in the local setting so, this is not just random association and possible explanation was done in the discussion section.
3. Methodology: Need more explanation on the tool you have used and its validity. Any specific reason to select only those variables in your study. Its not clear how you addressed your confounders
Revised: The tool was adapted from the previous study by Sharma SR et al. and other similar studies done in Nepal. The tool was translated into the Nepali language and pretested in Dhulikhel municipality; of Kavrepalanchok District among 10 percent of sample size i.e. 12 cases and 24 controls.
Response: Possible confounders were addressed by randomization and multivariate analysis.
4. Result: Some of your finding to do not meet the principle of Biological Plausibility
Response: The Biological plausibility of each finding from the multivariate analysis was explained in the discussion section.
Reviewer #2:
Note: Please find all the comments in the attached reviewed manuscript file in track change format.
Response: All the comments in the track change format are addressed individually.
Title of research: Clear
Abstract:
Organised well.
Line 28 and 29: Variables are not clear. This needs to be more specific like other variables. Suggestion: Not having a kitchen in the same living room.
Response: the variable location of kitchen wants to know about the kitchen in the same living house or separate from the living house. So it has been explained as having a kitchen in the same living house.
Line 33-35: In conclusion This seems recommendations. Please mention your conclusion based on your objective of this research only.
Revised: Having the kitchen in the same living house, iron intake less than 180 tablets during pregnancy, maternal weight gain less than 6.53 kg during the second and third trimester, co-morbidity during pregnancy and preterm birth were the risk factors associated with low birth weight.
Introduction:
I would suggest adding few literatures from Nepal as well.
Intext citation was added to highlight the literature that was reviewed from Nepal.
Methodology:
Line 54: I think this word (retrospective) is not required here.
Removed: An unmatched case-control study was used.
Line 56: Better to include health institutions.
Response: The data were taken from 31 health institutions of Dang district so it difficult to include the name of the health institution.
Revised: The study population was mothers, who delivered their babies in the governmental health institutions (28 birthing centers and 3 hospitals) of Dang from 17th August to 16th November 2018.
Line 67: Start from word…not from number.
Revised: One hundred and twenty-three
Line 68: Not clear….How did you select? Please be specific.
Added: One hundred and twenty-three cases were selected from the list of 224 cases and 246 controls from the list of 777 controls randomly independent of exposure status by generating random numbers The 123 cases and 246 controls with the highest random number were visited with the help of FCHV, local leaders, teachers for the data collection.
Results:
Line 88: Table 1: Rectify the included variables and better to concise the writing.
It means the kitchen is within the sleeping room in a house. What is the rationale of this category?
The variable wants to know whether the participants had a separate kitchen or not.
The rationale behind 6.53 Kg is, in our context it is difficult to know the exact weight gain during the pregnancy, however the weight gain in each ANC visit was recorded in both ANC card and maternity and newborn register. As per the study done in Low and middle-income county by Diane Coffey in 2015, concluded that the weight gain between the second and third trimester was 6.53Kg and this information can be easily accessible from ANC cards as well as maternity register.

	Line 90-104: Add the clear component of variables<br>Added: Added as per comments.<br>Line 105: Table 2: Is the p-value same for both bivariate and multivariate analysis? (As you can see there is a change in Confidence Interval in bivariate and multivariate analysis.)<br>The P-value is of the chi-square test.<br>In the age of mothers, 20-30 years category as a reference is more scientific.<br>The reference category changed to 20-30 years and analysis did accordingly.<br>Other variable needs to be more specific. (Suggested in track change attached manuscript file.)<br>The location of the kitchen is the proxy indicator of Indoor air pollution.<br>Smoking by a family member is the proxy indicator of second-hand smoking.<br>Smoking habit indicates the habit of smoking any form of tobacco; Ciggrate, hukka and other traditional forms of smoking like rolling of tobacco in green leaf and smoke.<br>Food frequency per day: It is recommended that pregnant women should take one additional meal during their pregnancy for healthy growth and development of a fetus. Based on this line, we can assume that when she had taken an adequate diet or not, however, we cannot say it is balanced or not. It reveals the quantitative prospect of diet than qualitative.<br>Line 106: Here you said both bivariate and multivariate analysis shown in the table but in your title of the table only multivariate included. I think you need to format the table<br>The table has been formatted.<br>Discussion:<br>Line 126-129: You need to change your reference age group for statistical analysis. I already mentioned above in your result section.<br>Revised: The reference age group has been changed and statistical analysis was done accordingly.<br>Line 131: Better to add in-text citation<br>Added<br>Line 147; Mention supporting evidence with in-text citation.<br>Intext citation with supporting evidence has been added.<br>Line 166: Is this only clinical assessment?<br>It was based on the diagnosis made by health workers (any level) by reviewing cards.<br>Line 172: Better to mention in-text citation.<br>Mentioned<br>Line 178: How randomly? Need a better explanation.<br>Explained in the methodological section<br>Conclusion:<br>Line 191-193: But how?? You need more justification.<br>The recommendation was rearranged.<br>References:<br>Better to add doi and PMID numbers if available.<br>Added where possible.
**Additional Information:**	
**Question**	**Response**
**Financial Disclosure**<br><br>Enter a financial disclosure statement that describes the sources of funding for the work included in this submission. Review the submission guidelines for detailed requirements. View published research articles from *PLOS ONE* for specific examples.<br><br>This statement is required for submission and **will appear in the published article** if the submission is accepted. Please make sure it is accurate.	The authors received no specific funding for this work.

**Unfunded studies**<br>Enter: *The author(s) received no specific funding for this work.*<br><br>**Funded studies**<br>Enter a statement with the following details:<br>• Initials of the authors who received each award<br>• Grant numbers awarded to each author<br>• The full name of each funder<br>• URL of each funder website<br>• Did the sponsors or funders play any role in the study design, data collection and analysis, decision to publish, or preparation of the manuscript?<br>• **NO** - Include this sentence at the end of your statement: *The funders had no role in study design, data collection and analysis, decision to publish, or preparation of the manuscript.*<br>• **YES** - Specify the role(s) played.<br><br>* typeset	
**Competing Interests**<br><br>Use the instructions below to enter a competing interest statement for this submission. On behalf of all authors, disclose any competing interests that could be perceived to bias this work—acknowledging all financial support and any other relevant financial or non-financial competing interests.<br><br>This statement **will appear in the published article** if the submission is accepted. Please make sure it is accurate. View published research articles from *PLOS ONE* for specific examples.	The authors have declared that no competing interests exist.

Powered by Editorial Manager® and ProduXion Manager® from Aries Systems Corporation

**NO authors have competing interests**<br><br>Enter: *The authors have declared that no competing interests exist.*<br><br>**Authors with competing interests**<br><br>Enter competing interest details beginning with this statement:<br><br>*I have read the journal's policy and the authors of this manuscript have the following competing interests: [insert competing interests here]*<br><br>* typeset	
**Ethics Statement**<br><br>Enter an ethics statement for this submission. This statement is required if the study involved:<br><br>• Human participants<br>• Human specimens or tissue<br>• Vertebrate animals or cephalopods<br>• Vertebrate embryos or tissues<br>• Field research<br><br>Write "N/A" if the submission does not require an ethics statement.<br><br>General guidance is provided below. Consult the submission guidelines for detailed instructions. **Make sure that all information entered here is included in the Methods section of the manuscript.**	This study was approved by Institutional Review Committee (IRC) of Institute of Medicine (IOM) Tribhuvan University.<br>Letter number 69(6-11-E)2/075/076 had stated "I am pleased to inform you that after carefully evaluation, the above mentioned research proposal has been approved by Institutional Review Committee (IRC) of Institute of Medicine (IOM) Tribhuvan University on August 19, 2018."<br>Written consent was obtained from research participants.

**Format for specific study types**<br><br>**Human Subject Research (involving human participants and/or tissue)**<br>• Give the name of the institutional review board or ethics committee that approved the study<br>• Include the approval number and/or a statement indicating approval of this research<br>• Indicate the form of consent obtained (written/oral) or the reason that consent was not obtained (e.g. the data were analyzed anonymously)<br><br>**Animal Research (involving vertebrate animals, embryos or tissues)**<br>• Provide the name of the Institutional Animal Care and Use Committee (IACUC) or other relevant ethics board that reviewed the study protocol, and indicate whether they approved this research or granted a formal waiver of ethical approval<br>• Include an approval number if one was obtained<br>• If the study involved *non-human primates*, add *additional details* about animal welfare and steps taken to ameliorate suffering<br>• If anesthesia, euthanasia, or any kind of animal sacrifice is part of the study, include briefly which substances and/or methods were applied<br><br>**Field Research**<br><br>Include the following details if this study involves the collection of plant, animal, or other materials from a natural setting:<br>• Field permit number<br>• Name of the institution or relevant body that granted permission	
**Data Availability**<br><br>Authors are required to make all data underlying the findings described fully available, without restriction, and from the time of publication. PLOS allows rare exceptions to address legal and ethical concerns. See the PLOS Data Policy and FAQ for detailed information.	No - some restrictions will apply

A Data Availability Statement describing where the data can be found is required at submission. Your answers to this question constitute the Data Availability Statement and **will be published in the article**, if accepted.<br><br>**Important:** Stating 'data available on request from the author' is not sufficient. If your data are only available upon request, select 'No' for the first question and explain your exceptional situation in the text box.<br><br>Do the authors confirm that all data underlying the findings described in their manuscript are fully available without restriction?	
**Describe where the data may be found in full sentences. If you are copying our sample text, replace any instances of XXX with the appropriate details.**<br><br>• If the data are **held or will be held in a public repository**, include URLs, accession numbers or DOIs. If this information will only be available after acceptance, indicate this by ticking the box below. For example: *All XXX files are available from the XXX database (accession number(s) XXX, XXX.).*<br>• If the data are all contained **within the manuscript and/or Supporting Information files**, enter the following: *All relevant data are within the manuscript and its Supporting Information files.*<br>• If neither of these applies but you are able to provide **details of access elsewhere**, with or without limitations, please do so. For example:<br><br>*Data cannot be shared publicly because of [XXX]. Data are available from the XXX Institutional Data Access / Ethics Committee (contact via XXX) for researchers who meet the criteria for access to confidential data.*<br><br>*The data underlying the results presented in the study are available from (include the name of the third party*	All relevant data are within the manuscript and its supporting information files.

and contact information or URL).<br>• This text is appropriate if the data are owned by a third party and authors do not have permission to share the data.<br><br>* typeset	
Additional data availability information:	

Powered by Editorial Manager® and ProduXion Manager® from Aries Systems Corporation

Manuscript Click here to access/download;Manuscript;Manuscript.docx

Low birth weight and its associated risk factors: health facility-based case-control study

Anil K.C.[1*], Prem Lal Basel[2], Sarswoti Singh[3]

[1] Health Foundation Nepal

[2] Tribhuvan University Teaching Hospital, Department of Community Medicine and Public health

[3] Tribhuvan University Teaching Hospital, Department of Community Medicine and Public health

* Corresponding author

E-mail: kcanil707@gmail.com

Abstract

Background: Low birth weight is a preventable public health problem. It is an important determinant of child survival and development, as well as long-term consequences like the onset of non-communicable disease in the life course. A large number of mortality and

morbidity can be prevented by addressing the factors associated with low birth weight. The main objective of this study was to identify associated risk factors of low birth weight.

Methodology: A health facility-based unmatched case-control study was carried out from July 2018 to March 2019 among the mothers who delivered in health facilities of Dang district of Nepal from 17th August to 16th November 2018. The total sample size for the study was 369; 123 cases and 246 controls. Cases and controls were randomly selected independent of the exposure status in the ratio of 1:2. Information regarding exposure status was assessed through interviews and medical records. Mothers who delivered outside Dang districts were excluded from the study. Ethical clearance was obtained from the Institutional Review Committee (IRC) of the Institute of Medicine, Tribhuvan University and written consent was taken from each participant after explaining the objectives of the study.

Results: Multivariate logistic regression found that having the kitchen in the same living house (AOR 2.7, CI: 1.5-4.8), iron intake less than 180 tablets (AOR 3.2, CI: 1.7-5.7), maternal weight gain during second and third trimester less than 6.53 kg (AOR 2.6, CI: 1.5-4.7), co-morbidity during pregnancy (AOR 2.4, CI: 1.3-4.5), preterm birth (AOR 2.9, CI: 1.4-6.1) were the risk factors associated with low birth weight.

Conclusion: Having the kitchen in the same living house, iron intake less than 180 tablets during pregnancy, maternal weight gain less than 6.53 kg during the second and third trimester, co-morbidity during pregnancy and preterm birth were the risk factors associated with low birth weight.

Introduction

World Health Organization defines low birth weight (LBW) as the birth weight less than 2500 grams irrespective of gestational age (1). LBW is a valuable public health indicator of maternal health, nutrition, healthcare delivery, and poverty as LBW babies are at a higher risk of death and illness shortly after birth and non-communicable disease in the life course (2). LBW infants are 20 times more likely to develop complications and die in comparison to normal weight babies (3). LBW babies are in the potential risk of cognitive deficits, motor delays, cerebral palsy, and other behavior and psychological problem (4-8). The household cost, as well as health system costs, could be saved by reducing the burden of LBW (9). The pathophysiology of low birth weight is unclear, whereas intrauterine growth retardation (IUGR) and preterm birth considered as the caus[illegible]f LBW (3). IUGR is the outcome of insufficient uterine–placental perfusion and fetal nutrition affecting the overall anthropometric parameter of the fetus. IUGR newborn has typical features of malnutrition. Extra-uterine infection, trauma, illness, IUGR, fetal infection, and anomalies are the contributing factors for preterm birth, resulting in growth retardation which ultimately results in LBW(10, 11). LBW is considered a significant public health problem as it is estimated that 15% to 20% of all birth worldwide are LBW. The prevalence of LBW varies across regions with the highest 28% in South Asia and the lowest 6% in East Asia and the Pacific region (12). The prevalence of LBW in Nepal ranges from 12% to 21.6%, (13-15). A few descriptive and hospital-based case-control studies have been done in Nepal(14-19). These descriptive and hospital-based studies could not represent the scenario of risk factors of LBW at the community level. Hence this study aims to identify the scenario of associated risk factors of LBW at the community level.

Methodology

Ethical clearance was obtained from the Institutional Review Committee (IRC) of the Institute of Medicine, Tribhuvan University. Permission was taken from the District Public Health Office (DPHO) Dang and respective health facilities. Written consent was taken from each participant after explaining the objectives of the study. After the interview, the mothers were informed about the importance of growth monitoring, exclusive breastfeeding, immunization and appropriate time of weaning.

An unmatched case-control study was used. This study was conducted in Dang district of Nepal. This study was approved by the Institutional Review Committee (IRC) of Institute of Medicine (IOM) Tribhuvan University on August 19, 2018. The study population was mothers, who delivered their babies in the governmental health institutions (28 birthing centers and 3 hospitals) of Dang from 17th August to 16th November 2018. The study population was divided into case and control as per the following definition.

Case: Mother delivering singleton live-born baby with birth weight less than 2500 grams without any congenital anomalies and were originally from Dang district.

Control: Mother delivering singleton live-born baby with birth weight more or equal to 2500 grams without any congenital anomalies and were from the same Dang district.

The sample size was calculated using EpiInfo software version 7. This was calculated by taking power at 80%, confidence level as 95%, the percentage of control exposed as 65.40, the odds ratio of 2.06 from the maternal weight against LBW (20) and the ratio of case to control was 1:2. The total sample size was 369 with 123 cases and 246 controls. The eligible numbers of participants were enlisted from the maternal and neonate health register of 28 birthing centers and 3 hospitals of Dang District. One hundred and twenty-three cases were

selected from the list of 224 cases and 246 controls from the list of 777 controls randomly independent of exposure status by generating random numbers. The 123 cases and 246 controls with the highest random number were visited with the help of FCHV, local leaders, teachers for the data collection. Face to face interview was done with the participants for the collection of data using a semi-structured questionnaire. Information regarding weight gain, age, ANC visit, birth weight, comorbidity, gestational age etc was taken through reviewing Antenatal Care (ANC) card and Maternal and newborn register to avoid possible recall bias. The tool was adapted from the previous study by Sharma SR et al. and other similar studies done in Nepal. The tool was translated into the Nepali language and pretested in Dhulikhel municipality; of Kavrepalanchok District among 10 percent of sample size i.e. 12 cases and 24 controls.

Data entry was done in Epi data Version 3.1 following coding. Data analysis was done using SPSS software version 21. Bivariate associations between independent variables and low birth weight were tested through the Chi-square test and the association was analyzed by calculating crude odds ratios (OR) at 95% confidence interval through binary logistic regression. Multivariate logistic regression was examined for the relationship between independent variables and low birth weight to address the confounding effect. Hosmer and Lemeshow test was used to test the goodness-of-fit for regression models. The test statistic was 0.69 ($p > 0.05$) that showed that the model adequately fit the data.

Results

The mean age of the participants was 23 years (SD 4.4 years);

Table 1: Distribution of participants according to socio-demographic, maternal factors and co-morbidity during pregnancy

Variables	Case (123)	Control (246)	Total (369)
	n (%)	n (%)	
Age of mother			
<20 Years	35 (28.5%)	55 (22.4%)	90 (24.4%)
20-30 Years	79 (64.2%)	178 (72.4%)	257 (69.6%)
>30 years	9 (7.3%)	13 (5.3%)	22 (6.0%)
Support from husband in day to day activities			
Yes	102 (82.9%)	222 (90.2%)	324 (87.8%)
No	21 (17.1%)	24 (9.8%)	45 (12.2%)
Cooking material			
Fire wood and Kerosene	87 (70.7%)	158 (64.2%)	245 (66.4%)
LPG and Bio Gas	36 (29.3%)	88 (35.8%)	124 (33.6%)
Location of kitchen			
Same house	86 (69.9%)	110 (44.7%)	196 (53.1%)
Separate house and outside	37 (30.1%)	136 (55.3%)	173 (46.9%)
Smoking by family member			
Yes	65 (52.8%)	100 (40.7%)	165 (44.7%)
No	58 (47.2%)	146 (59.3%)	204 (55.3%)
Smoking habit			
Yes	6 (4.9%)	2 (.8%)	8 (2.2%)
No	117 (95.1%)	244 (99.2%)	361 (97.8%)
Food frequency per day			
Twice	30 (24.4%)	44 (17.9%)	74 (20.1%)
Thrice	81 (65.9%)	166 (67.5%)	247 (67%)
More	12 (9.6%)	36 (14.6%)	48 (13.0%)
Type of food use			
As usual	56 (45.5%)	76 (30.9%)	132 (35.8%)
Addition food (Any group)	67 (54.5%)	170 (69.1%)	237 (64.2%)
ANC visit as per protocol			
Yes	80 (65.0%)	208 (84.6%)	288 (78.0%)
No	43 (35.0%)	38 (15.4%)	81 (22.0%)
Number of iron tablets used			
<180	60 (48.8%)	40 (16.3%)	100 (27.1%)
180 and more	63 (51.1%)	206 (83.7%)	269 (72.9%)
Weight gain between second and third trimester			
<6.53 kilogram (kg)	91 (74.0%)	120 (48.8%)	211 (57.2%)
6.53 kg and above	32 (26.0%)	126 (51.2%)	158 (42.8%)
Number of children			
1 (Primiparity)	79 (64.2%)	121 (49.2%)	200 (54.2%)

2	32 (26.0%)	93 (37.8%)	125 (33.9%)
3	8 (6.5%)	26 (10.6%)	34 (9.2%)
4 and above	4 (3.3%)	6 (2.4%)	10 (2.7%)
Health problem			
Yes	50 (40.7%)	34 (13.8%)	84 (22.8%)
No	73 (59.3%)	212 (86.2%)	285 (77.2%)
Preterm birth			
Yes	33 (26.8%)	21 (8.5%)	54 (14.6%)
No	90 (73.2%)	225 (91.5%)	315 (85.4%)

Table 1 depicts that getting support from their husbands in day to day activities during pregnancy was quite con[illegible]n. The major (66.4%) fuel used during cooking was firewood and kerosene. The majority (53.1%) of the household did not have a separate kitchen. Majority (55.3%) of participants' family members did not smoke any form of cigarette. A small portion (2.2%) of participants had the habit of smoking cigarettes during pregnancy. Two-third (67%) of participants had their meal thrice a day and 64.2% had included additional food groups in their meal at the time of pregnancy. Majority (78%) of participants had attended ANC visit as per the protocol of the government of Nepal. Similarly, 73% of participants had taken 180 or more iron tablets during pregnancy. More than half of the participants had gained weight less than 6.53 kg during the second and third trimester. Fifty-four percentages of participants had one child. Majority (59.3% among cases and 86.2% among controls) of the research participants did not face any health problems (co-morbidities) during their pregnancy and 14.6% of the babies were born before 37 weeks of gestation.

Table 2: Bivariate and Multivariate analysis of independent variables against low birth weight

Variables	p-value	Crude OR	CI (95%)	Adjusted OR	CI (95%)
Age of mother					
<20 Years	0.4	1.4	0.9 to 2.4	0.8	0.4 to 1.6
>30 years		1.6	0.6 to 3.8	1.5	0.4 to 5.3
20-30 Years		Ref		Ref	

Support from husband in day to day activities					
No	0.04	1.9	1.0 to 3.6	1.5	0.7 to 3.3
Yes		Ref		Ref	
Cooking material					
Fire wood and Kerosene	0.2	1.4	0.8 to 2.2	1.4	0.7 to 2.6
LPG and Bio Gas		Ref		Ref	
Location of kitchen (Proxy of indoor air pollution)					
Same house	<0.0001	2.9	1.8 to 4.6	2.7	1.5 to 4.8
Separate house and outside		Ref		Ref	
Smoking by family member					
Yes	0.026	1.6	1.1 to 2.5	1.4	0.8 to 2.5
No		Ref		Ref	
Smoking habit					
Yes	0.032	6.3	1.2 to 31.5	5.3	0.7 to 42.7
No		Ref		Ref	
Food frequency per day					
Twice	0.19	2	0.9 to 4.6	1.3	0.5 to 3.6
Thrice		1.5	0.7 to 3	1.5	0.7 to 3.5
More		Ref		Ref	
Type of food use					
As usual	0.006	1.9	1.2 to 2.9	0.9	0.5 to 1.6
Addition food (Any group)		Ref		Ref	
ANC visit as per the protocol of Nepal Government					
No	<0.0001	2.9	1.8 to 4.9	1.7	0.9 to 3.2
Yes		Ref		Ref	
Number of iron tablets used					
<180	<0.0001	4.8	3.0 to 7.9	3.2	1.7 to 5.7
180 and more		Ref		Ref	
Weight gain					
Less than 6.53 kg	< 0.0001	3	1.9 to 4.8	2.6	1.5 to 4.7
6.53 kg and above		Ref		Ref	
Number of children					
1 (Primiparity)	0.03	1.9	1.2 to 3.1	1.8	0.9 to 3.6
3		0.9	0.4 to 2.2	0.4	0.1 to 1.2
4 and above		1.9	0.5 to 7.3	0.6	0.1 to 3.8
2		Ref		Ref	
Health problem (Co-morbidity)					
Yes	<0.0001	4.3	2.6 to 7.1	2.4	1.3 to 4.5
No		Ref		Ref	
Preterm birth					
Yes	<0.0001	3.9	2.2 to 7.2	2.9	1.4 to 6.1
No		Ref		Ref	

Table 2 shows the bivariate and multivariate analysis of dependent and independent variables. In bivariate analysis; support from husband during pregnancy, use of firewood and kerosene during cooking, having kitchen in the same living house, cigarette smoking by family members, cigarette smoking by mother during pregnancy, use of additional food groups in their diet during pregnancy, four ANC visit as per protocol of Government of Nepal, iron tablets intake less than 180 tablets during pregnancy, weight gain during pregnancy less than 6.53 kg in-between second and third trimester, mother delivering her first baby, health problem during pregnancy and preterm baby were associated with low birth weight.

In multivariate analysis, having kitchen in the same living house (AOR 2.7, CI: 1.5-4.8), Iron intake less than 180 tablets (AOR 3.2, CI: 1.7-5.7), maternal weight gain less than 6.53 kg during second and third trimester (AOR 2.6, CI: 1.5-4.7), co-morbidities during pregnancy (AOR 2.4, CI: 1.3- 4.5) and preterm birth (AOR 2.9, CI: 1.4-6.1) were significantly associated at 95% confidence interval with the low birth weight. Similarly, age of mother, support from husband during pregnancy, use of firewood and kerosene during cooking, smoking habit of the mother, smoking by family member, food frequency less than three per day, use of any additional food group during pregnancy, four ANC visit as per protocol, first children was not associated with LBW in this study.

Discussion

This study analyzed the socio-demographic factors, maternal factors, and co-morbidities during recent pregnancy against low birth weight during delivery.

The maternal age is considered as a key factor for the healthy outcome of pregnancy. This study revealed no statistical association between maternal age and low birth weight which contradicts with the study done in Nepal, that shows a higher risk of delivering low birth weight babies by mother age less than 20 and more than 30 years (16, 17, 21). Smoking during pregnancy had a negative effect on the growth and development of the fetus because of chemical substances present in it. Nicotine present in the cigarette cause vasoconstriction resulting in the low oxygen flow to the fetus and Carbon-monoxide forms carboxyhemoglobin which inhibits the oxygen release to fetal tissues(22) In bivariate analysis mother habit of smoking had a higher risk of low birth weight in reference to the mother who did not smoke a cigarette (OR 6.3, 95% CI: 1.2-31.5). This finding is consistent with the findings of similar studies done in Bangladesh and Turkey (23, 24). Though there was a risk, however, there was no significant association between smoking and low birth weight in multivariate analysis. This could be explained probably due to the small number of smokers in the study population. Moreover, it can also be explained by the social desirability bias, induced due to social stigma.

This study identified the location of the kitchen in the living house, iron intake less than 180 tablets, weight gain less than 6.53 kg during the second and third trimester, comorbidity during pregnancy, and preterm birth as the risk factors for low birth weight. The finding reveals that the cooking fuels namely firewood and kerosene use had a risk for LBW with reference to LPG and Biogas however, it was not statistically significant. This finding contradicts to the find of the study done by Kadam YR et al. and Washam C (25, 26), however, this study revealed that having a kitchen in the same living house (proxy of indoor air pollution) had 2.5 times higher risk of delivering low birth weight which may be due to,

living in the same house had higher risk and duration of exposure to the pollutants like $PM_{2.5}$, PM_{10}, NO_2, SO_2, CO caused by burning of fuels, leading to the impaired supply of oxygen, nutrition to the fetus resulting in the negative impact on the growth and development of fetus(27, 28). The amount of exposure was not measured quantitatively in this study.

This study showed that total iron tablet intake during pregnancy was associated with the birth weight of the child. Mothers who took less than 180 tablets of iron during their pregnancy were three times more likely to deliver low birth weight babies with reference to mothers who took iron equal to or more than 180 tablets during their pregnancy period (AOR 3.2, CI: 1.7-5.8). This finding is similar to the studies conducted in Nepal (16, 29). Low iron tablets intake causes the poor delivery of iron to the fetus thereby impair in proper hormonal and neuronal regulation of pregnancy and poor oxygenation to the fetus leading to the poor growth and development of the fetus (30). However, the iron intake through diet during pregnancy was not measured in both cases and control.

The minimum standard weight gain during the second and third trimester is set as 6.53 kg (31). Women who gained weight less than 6.53 kg during the second and third trimester had 3 times higher risk of delivering low birth weight baby with reference to women whose weight gain was 6.53 kg or above (AOR 2.8, CI: 1.6-5.0). This finding is similar to the study done in Bangladesh (32) and Mozambique (33). The weight gain during pregnancy is impaired due to ill health, poor sanitation, and inadequate balance diet which at the end hamper the proper growth and development of the baby.

Women who had at least one health problem during their pregnancy were at higher risk of delivering low birth weight in comparison to women without any health problem (AOR 2.6, CI: 1.4-4.8). This finding is consistent with the study done in Nepal (17, 20). Likewise, this

study suggest that mother delivering baby before completion of 37 weeks of gestation had higher risk of delivering low birth weight than the mothers who deliver the term baby (AOR 2.6, CI: 1.2-5.5) which is in line with the study done in Nepal (17), Ethiopia (34) and Kenya (35). Biologically it can be explained that preterm birth was less likely to get sufficient time for maturity, growth and nutrient intake which therefore can lead to low birth weight(36).

In this study, the researcher has retrieved maternal information namely gestational weight, iron tablets intake, gestational age, co-morbidity, frequency of ANC visits and birth weight of a baby from ANC card and maternity register to limit the recall bias. The selection of cases and controls were based on the records of maternal and neonatal register therefore, it is less likely that this study has misclassification biases both in the exposure and case-control categories. Controls were selected randomly independent of the exposure status and moreover, as there was no non-responses rate in both the group, it is less likely that this study would suffer from selection biases.

Conclusion and recommendation

This study concluded that the having the kitchen in the same living house (proxy of indoor air pollution), iron intake less than 180 tablets during pregnancy, weight gain less than 6.35 kg during the second and third trimester, co-morbidity during pregnancy, and preterm delivery were found to be associated risk factors of low birth weight. Thus, identified risk factors can be efficiently prevented through small doable actions that a family can apply and the mother can easily carry out. Maternal health programs can be directed towards motivating and tracking pregnant mothers for complete iron tablets intake during her pregnancy. Intake of balance diet as per the protocol of the Government of Nepal for healthy growth and

development of the child inside the uterus is of paramount importance. Family should help the mother for adequate rest, nutrition and healthy behavior to prevent risk factors identified in this study.

Acknowledgment

Authors would like to thank Mr. Keshav Raj Pandit, Senior Public Health Administrator and the entire staff of DPHO, Dang for their kind support in the coordination with health facilities, as well as to Female Community Health Volunteers, local leaders and teachers for their support during data collection. The authors would also like to express sincere thanks to all research participants for their valuable time and information.

Supporting information

All data files are available from the Figshare database

https://doi.org/10.6084/m9.figshare.11984433.v1

Reference

1. International Statistical Classification of Diseases and Related Health Problems- 10. Disorders related to short gestation and low birth weight, not elsewhere classified: World Health Organization; 2016.
2. Agbozo F, Abubakari A, Der J, Jahn A. Prevalence of low birth weight, macrosomia and stillbirth and their relationship to associated maternal risk factors in Hohoe Municipality, Ghana. Midwifery. 2016;40:200-6.
3. UNICEF, WHO. Low Birth Weight: Country, regional and global estimates. UNICEF, New York, 2004.
4. Mathewson KJ, Chow CH, Dobson KG, Pope EI, Schmidt LA, Van Lieshout RJ. Mental health of extremely low birth weight survivors: A systematic review and meta-analysis. Psychological bulletin. 2017;143(4):347-83.

5. Al Hazzani F, Al-Alaiyan S, Hassanein J, Khadawardi E. Short-term outcome of very low-birth-weight infants in a tertiary care hospital in Saudi Arabia. Annals of Saudi Medicine. 2011;31(6):581-5.
6. Chang H-Y, Sung Y-H, Wang S-M, Lung H-L, Chang J-H, Hsu C-H, et al. Short- and Long-Term Outcomes in Very Low Birth Weight Infants with Admission Hypothermia. PloS one. 2015;10(7):e0131976.
7. Fitzgibbons SC, Ching Y, Yu D, Carpenter J, Kenny M, Weldon C, et al. Mortality of necrotizing enterocolitis expressed by birth weight categories. Journal of Pediatric Surgery.44(6):1072-6.
8. Fan RG, Portuguez MW, Nunes ML. Cognition, behavior and social competence of preterm low birth weight children at school age. Clinics. 2013;68(7):915-21.
9. Sicuri E, Bardají A, Sigauque B, Maixenchs M, Nhacolo A, Nhalungo D, et al. Costs Associated with Low Birth Weight in a Rural Area of Southern Mozambique. PloS one. 2011;6(12):e28744.
10. Sharma D, Shastri S, Sharma P. Intrauterine Growth Restriction: Antenatal and Postnatal Aspects. Clinical medicine insights Pediatrics. 2016;10:67-83.
11. Cutland CL, Lackritz EM, Mallett-Moore T, Bardaji A, Chandrasekaran R, Lahariya C, et al. Low birth weight: Case definition & guidelines for data collection, analysis, and presentation of maternal immunization safety data. Vaccine. 2017;35(48 Pt A):6492-500.
12. Global Nutrition Targets 2025: Low Birth Weight Policy Brief. World Health Organization, 2014.
13. Ministry of Health Nepal, New ERA, and, ICF. Nepal Demographic and Health Survey 2016. Kathmandu, Nepal: Ministry of Health, New ERA, ICF International; 2017.
14. Malla M, Joshi DR, Chhetri K, Pandey P. Prevalence and contributing factors of low birth weight babies in institutional delvery. Journal of Patan Academy of Health Sciences. 2015;2(1):4.
15. Prajapati R, Shrestha S, Bhandari N. Prevalence and Associated Factors of Low Birth Weightamong Newborns in a Tertiary Level Hospital in Nepal. Kathmandu University Medical Journal. 2018;16(61):4.
16. Karuna Laxmi Shakya NS, Amod Kumar Poudyal, Rajendra Prasad Koju, Peter Oyloe and Sharad Raj Onta. Key factors associated with low birth weight at term in Nepal: A Case Control study. International Journal of Clinical and Biomedical Research. 2015;1(2):8.
17. Sharma SR, Giri S, Timalsina U, Bhandari SS, Basyal B, Wagle K, et al. Low birth weight at term and its determinants in a tertiary hospital of Nepal: a case-control study. PloS one. 2015;10(4):e0123962.
18. Yadav DK, Chaudhary U, Shrestha N. Risk factors associated with low birth weight. Journal of Nepal Health Research Council. 2011;9(2):159-64.
19. Kandel KP, Kafle S. Risk Factors Associated with Low Birth Weight among Deliveries at Bharatpur Hospital. Journal of Nepal Health Research Council. 2017;15(2):169-73.
20. Bhaskar RK, Deo KK, Neupane U, Chaudhary Bhaskar S, Yadav BK, Pokharel HP, et al. A Case Control Study on Risk Factors Associated with Low Birth Weight Babies in Eastern Nepal. International journal of pediatrics. 2015;2015:807373.
21. Yadav DK, Chaudhary U, Shrestha N. Risk Factors Associated with Low Birth Weight. Nepal Health Research Council. 2011;9(19):159-64.
22. Lambers DS, Clark KE. The maternal and fetal physiologic effects of nicotine. Seminars in Perinatology. 1996;20(2):115-26.

23. Hosain GM, Chatterjee N, Begum A, Saha SC. Factors associated with low birthweight in rural Bangladesh. Journal of tropical pediatrics. 2006;52(2):87-91.
24. Atessahin E, Pirincci E. Risk Factors Associated with Low Birth Weight Infants Born in Elazig, Eastern of Turkey. Iranian journal of public health. 2015;44(9):1299-300.
25. Kadam YR, Mimansa A, Chavan PV, Gore AD. Effect of prenatal exposure to kitchen fuel on birth weight. Indian journal of community medicine : official publication of Indian Association of Preventive & Social Medicine. 2013;38(4):212-6.
26. Washam C. Cooking with wood may fuel low birth weight: kitchen smoke puts babies at risk. Environmental health perspectives. 2008;116(4):A173-A.
27. Boy E, Bruce N, Delgado H. Birth weight and exposure to kitchen wood smoke during pregnancy in rural Guatemala. Environmental health perspectives. 2002;110(1):109-14.
28. Oliveira BFAd, Ignotti E, Hacon SS. A systematic review of the physical and chemical characteristics of pollutants from biomass burning and combustion of fossil fuels and health effects in Brazil. Cadernos de Saúde Pública. 2011;27:1678-98.
29. Bondevik GT, Lie RT, Ulstein M, Kvåle G. Maternal hematological status and risk of low birth weight and preterm delivery in Nepal. Acta Obstetricia et Gynecologica Scandinavica. 2001;80(5):402-8.
30. Beard JL. Micronutrient Deficiencies in the First Months of Life: Karger Publishers; 2004.
31. Coffey D. Prepregnancy body mass and weight gain during pregnancy in India and sub-Saharan Africa. Proceedings of the National Academy of Sciences. 2015;112(11):3302-7.
32. Hassoune S, Bassel S, Nani S, Elbouri H, Zine K, Maaroufi A. [Maternal factors associated with low birth weight: case-control study in a Moroccan public hospital]. The Pan African medical journal. 2015;20:303.
33. Osman N, Challis K, Cotiro M, Nordahl G, Berström S. Perinatal outcome in an obstetric cohort of Mozambican women. Journal of tropical pediatrics. 2001;47(1):30-8.
34. Gebremedhin M, Ambaw F, Admassu E, Berhane H. Maternal associated factors of low birth weight: a hospital based cross-sectional mixed study in Tigray, Northern Ethiopia. BMC pregnancy and childbirth. 2015;15:222.
35. Muchemi OM, Echoka E, Makokha A. Factors associated with low birth weight among neonates born at Olkalou District Hospital, Central Region, Kenya. The Pan African medical journal. 2015;20:108.
36. Negrato CA, Gomes MB. Low birth weight: causes and consequences. Diabetology & Metabolic Syndrome. 2013;5(1):49.

Click here to access/download
Supporting Information
Supporting information.sav

Low birth weight and its associated risk factors: health facility-based case-control study

Anil K.C.[1*], Prem Lal Basel[2], Sarswoti Singh[3]

[1] Health Foundation Nepal

[2] Tribhuvan University Teaching Hospital, Department of Community Medicine and Public health

[3] Tribhuvan University Teaching Hospital, Department of Community Medicine and Public health

* Corresponding author

E-mail: kcanil707@gmail.com

Abstract

Background: Low birth weight is a preventable public health problem. It is an important determinant of child survival and development, as well as long-term consequences like the onset of non-communicable disease in the life course. A large number of mortality and

morbidity can be prevented by addressing the factors associated with low birth weight. The main objective of this study was to identify associated risk factors of low birth weight.

Methodology: A health facility-based unmatched case-control study was carried out from July 2018 to March 2019 among the mothers who delivered in health facilities of Dang district of Nepal from 17th August to 16th November 2018. The total sample size for the study was 369; 123 cases and 246 controls. Cases and controls were randomly selected independent of the exposure status in the ratio of 1:2. Information regarding exposure status was assessed through interviews and medical records. Mothers who delivered outside Dang districts were excluded from the study. Ethical clearance was obtained from the Institutional Review Committee (IRC) of the Institute of Medicine, Tribhuvan University and written consent was taken from each participant after explaining the objectives of the study.

Results: Multivariate logistic regression found that ~~the location of~~ having the kitchen in the same living house (AOR 2.7, CI: 1.5-4.8), iron intake less than 180 tablets (AOR 3.2, CI: 1.7-5.7), maternal weight gain during second and third trimester less than 6.53 kg (AOR 2.6, CI: 1.5-4.7), co-morbidity during pregnancy (AOR 2.4, CI: 1.3-4.5), preterm birth (AOR 2.9, CI: 1.4-6.1) were the risk factors associated with low birth weight.

Conclusion: ~~The identified risk factors can be efficiently eliminated through behavior change communication programs focused on small doable actions that a family can apply and the mother can easily carry out.~~Having the kitchen in the same living house, iron intake less than 180 tablets during pregnancy, maternal weight gain less than 6.53 kg during the second and third trimester, co-morbidity during pregnancy and preterm birth were the risk factors associated with low birth weight.

Introduction

World Health Organization defines low birth weight (LBW) as the birth weight less than 2500 grams irrespective of gestational age (1). LBW is considered as a a valuable public health indicator ofthat reflects maternal health, nutrition, healthcare delivery, and poverty as,. LBW babies are at a higher risk of death and illness shortly after birth and non-communicable disease in the life course (2). LBW infants are 20 times more likely to develop complications and die in compare comparison to normal weight babies (3). LBW babies are in the potential risk of cognitive deficits, motor delays, cerebral palsy, and other behavior and psychological problem (4-8). The household cost, as well as health system costs, could be saved by reducing the burden of LBW (9). The pathophysiology of low birth weight is unclear, whereas intrauterine growth retardation (IUGR) and preterm birth considered as the cause of LBW (3). IUGR is the outcome of insufficient uterine–placental perfusion and fetal nutrition affecting the overall anthropometric parameter of the fetus. IUGR newborn has typical features of malnutrition. Extra-uterine infection, trauma, illness, IUGR, fetal infection, and anomalies are the contributing factors for preterm birth, resulting in growth retardation which ultimately results in LBW(10, 11). LBW is considered a significant public health problem as it is estimated that 15% to 20% of all birth worldwide are LBW. The prevalence of LBW varies across regions with the highest 28% in South Asia and the lowest 6% in East Asia and the Pacific region (12). The prevalence of LBW in Nepal ranges from 12% to 21.6%, (13-15). A few descriptive and hospital-based case-control studies have been done in Nepal(14-19). These descriptive and hospital-based studies could not represent the scenarios of risk factors of LBW at the community level. Hence this study aims to identify the scenario of associated risk factors of LBW at the community level.

Methodology

Ethical clearance was obtained from the Institutional Review Committee (IRC) of the Institute of Medicine, Tribhuvan University. Permission was taken from the District Public Health Office (DPHO) Dang and respective health facilities. Written consent was taken from each participant after explaining the objectives of the study. After the interview, the mothers were informed about the importance of growth monitoring, exclusive breastfeeding, immunization and appropriate time of weaning.

A ~~retrospective~~ unmatched case-control study was used. This study was conducted in Dang District of Nepal. The study population was mothers ~~of children~~, who delivered their babies in the governmental health institutions (28 birthing centers and 3 hospitals) of Dang from 17th August to 16th November 2018. The study population was divided into case and control as per the following definition.

Case: Mother delivering singleton live-born baby with birth weight less than 2500 grams without any congenital anomalies and were originally from Dang district.

Control: Mother delivering singleton live-born baby with birth weight more or equal to 2500 grams without any congenital anomalies and were from the same Dang district.

The sample size was calculated using EpiInfo software version 7. This was calculated by taking power at 80%, ~~confident~~ confidence level as 95%, the percentage of control exposed as 65.40, the odds ratio of 2.06 from the maternal weight against LBW (20) and the ratio of case to control was 1:2. The total sample size was 369 with 123 cases and 246 controls. The eligible numbers of ~~research~~ participants were enlisted from the maternal and neonate health register of 28 birthing centers and 3 hospitals of Dang District. ~~123~~One hundred and twenty-

three cases were selected from the list of 224 cases and 246 controls from the list of 777 controls randomly independent of exposure status by generating random numbers. The 123 cases and 246 controls with the highest random number were visited with the help of FCHV, local leaders, teachers for the data collection. Face to face interview was done with the participants for the collection of data using a semi-structured questionnaire. Information regarding weight gain, age, ANC visit, birth weight, comorbidity, gestational age etc was taken through reviewing Antenatal Care (ANC) card and Maternal and newborn register to avoid possible recall bias. The tool was adapted from the previous study by Sharma SR et al. and other similar studies done in Nepal. Data were collected by scheduled interviews using semi-structured questionnaires, The tool was translated into the Nepali language and pretested in Dhulikhel municipality; of Kavrepalanchok District among 10 percent of sample size i.e. 12 cases and 24 controls. reviewing Antenatal Care (ANC) card and Maternal and newborn register.

Formatted: Font color: Auto

Data entry was done in Epi data Version 3.1 following coding. Data analysis was done using SPSS software version 21. Bivariate associations between independent variables and low birth weight were tested through the Chi-square test and the association was analyzed by calculating crude odds ratios (OR) at 95% confidence interval through binary logistic regression. Multivariate logistic regression was examined for the relationship between independent variables and low birth weight to address the confounding effect. Hosmer and Lemeshow test was used to test the goodness-of-fit for regression models. The test statistic was 0.69 ($p > 0.05$) that showed that the model adequately fit the data.

Ethical clearance was obtained from the Institutional Review Committee (IRC) of the Institute of Medicine, Tribhuvan University. Permission was taken from the District Public

Health Office (DPHO) Dang and respective health facilities. Written consent was taken from each participant after explaining the objectives of the study. After the interview, the mothers were informed about the importance of growth monitoring, exclusive breastfeeding, immunization and appropriate time of weaning.

Results

The mean age of the participants was 23 years (SD 4.4 years);

Table 1: Distribution of research participants according to socio-demographic, maternal factors and co-morbidity during pregnancy

Variables	Case (123) n (%)	Control (246) n (%)	Total (369)
Age of mother			
<20 Years	35 (28.5%)	55 (22.4%)	90 (24.4%)
20-30 Years	79 (64.2%)	178 (72.4%)	257 (69.6%)
>30 years	9 (7.3%)	13 (5.3%)	22 (6.0%)
Support from husband in day to day activities			
Yes	102 (82.9%)	222 (90.2%)	324 (87.8%)
No	21 (17.1%)	24 (9.8%)	45 (12.2%)
Cooking material			
Fire wood and Kerosene	87 (70.7%)	158 (64.2%)	245 (66.4%)
LPG and Bio Gas	36 (29.3%)	88 (35.8%)	124 (33.6%)
Location of kitchen			
Same house	86 (69.9%)	110 (44.7%)	196 (53.1%)
Separate house and outside	37 (30.1%)	136 (55.3%)	173 (46.9%)
Smoking by family member			
Yes	65 (52.8%)	100 (40.7%)	165 (44.7%)
No	58 (47.2%)	146 (59.3%)	204 (55.3%)
Smoking habit			
Yes	6 (4.9%)	2 (.8%)	8 (2.2%)
No	117 (95.1%)	244 (99.2%)	361 (97.8%)
Food frequency per day			
Twice	30 (24.4%)	44 (17.9%)	74 (20.1%)
Thrice	81 (65.9%)	166 (67.5%)	247 (67%)
More	12 (9.6%)	36 (14.6%)	48 (13.0%)

Type of food use			
As usual	56 (45.5%)	76 (30.9%)	132 (35.8%)
Addition food (Any group)	67 (54.5%)	170 (69.1%)	237 (64.2%)
ANC visit as per protocol			
Yes	80 (65.0%)	208 (84.6%)	288 (78.0%)
No	43 (35.0%)	38 (15.4%)	81 (22.0%)
Number of iron tablets used			
<180	60 (48.8%)	40 (16.3%)	100 (27.1%)
180 and more	63 (51.1%)	206 (83.7%)	269 (72.9%)
Weight gain between second and third trimester			
<6.53 kilogram (kg)	91 (74.0%)	120 (48.8%)	211 (57.2%)
6.53 kg and above	32 (26.0%)	126 (51.2%)	158 (42.8%)
Number of children			
1 (Primiparity)	79 (64.2%)	121 (49.2%)	200 (54.2%)
2	32 (26.0%)	93 (37.8%)	125 (33.9%)
3	8 (6.5%)	26 (10.6%)	34 (9.2%)
4 and above	4 (3.3%)	6 (2.4%)	10 (2.7%)
Health problem			
Yes	50 (40.7%)	34 (13.8%)	84 (22.8%)
No	73 (59.3%)	212 (86.2%)	285 (77.2%)
Preterm birth			
Yes	33 (26.8%)	21 (8.5%)	54 (14.6%)
No	90 (73.2%)	225 (91.5%)	315 (85.4%)

Table 1 depicts that ~~the research participants according to the socio-demographic, maternal factors and co-morbidity during pregnancy. The mean age of the research participants was 23 years (SD 4.4 years). G~~getting support from their husbands in day to day activities during pregnancy was quite common. The major (66.4%) fuel used during cooking was firewood and kerosene. The majority (53.1%) of the household did not have a separate kitchen. Majority (55.3%) of ~~the research~~ participants' family members did not smoke any form of cigarette. A small portion (2.2%) of ~~research~~ participants had the habit of smoking cigarettes during pregnancy. ~~More than half~~ Two-third (67%) of ~~the research~~ participants had their meal thrice a day and 64.2% had included additional food groups in their meal at the time of pregnancy. Majority (78%) of ~~the research~~ participants had attended ANC visit as per the protocol of the government of Nepal. Similarly, 73% of ~~the research~~ participants had taken

180 or more iron tablets during pregnancy. More than half of the ~~research~~ participants had gained weight less than 6.53 kg during the second and third trimester. Fifty-four percentages of ~~the research~~ participants had one child. Majority (59.3% among cases and 86.2% among controls) of the research participants did not face any health problems (co-morbidities) during their pregnancy and 14.6% of the babies were born before 37 weeks of gestation.

Table 2: Bivariate and Multivariate analysis of independent variables against low birth weight

Variables	~~χ2 (~~p-value~~)~~	Crude OR	CI (95%)	Adjusted OR	CI (95%~~)~~
Age of mother					
<20 Years	0.4	~~0.9~~1.4	~~0.4~~0.9 to ~~2.4~~2.4	~~0.5~~ 0.8	~~0.1~~0.4 to ~~2.2~~1.6
~~20-30 Years~~>30 years		~~0.6~~1.6	~~0.3~~0.6 to ~~1.6~~3.8	~~0.7~~ 1.5	~~0.2~~ 0.4 to ~~2.3~~ 5.3
~~>30 years~~20-30 Years		Ref		Ref	
Support from husband in day to day activities					
No	0.04	1.9	1.0 to 3.6	1.5	0.7 to 3.3
Yes		Ref		Ref	
Cooking material					
Fire wood and Kerosene	0.2	1.4	0.8 to 2.2	1.4	0.7 to 2.6
LPG and Bio Gas		Ref		Ref	
Location of kitchen (Proxy of indoor air pollution)					
Same house	<0.0001	2.9	1.8 to 4.6	2.7	1.5 to 4.8
Separate house and outside		Ref		Ref	
Smoking by family member					
Yes	0.026	1.6	1.1 to 2.5	1.4	0.8 to 2.5
No		Ref		Ref	
Smoking habit					
Yes	0.032	6.3	1.2 to 31.5	5.3	0.7 to 42.7
No		Ref		Ref	
Food frequency per day					
Twice	0.19	2	0.9 to 4.6	1.3	0.5 to 3.6
Thrice		1.5	0.7 to 3	1.5	0.7 to 3.5
More		Ref		Ref	
Type of food use					
As usual	0.006	1.9	1.2 to 2.9	0.9	0.5 to 1.6
Addition food (Any group)		Ref		Ref	
ANC visit as per the protocol of Nepal Government					

Formatted Table

No	<0.0001	2.9	1.8 to 4.9	1.7	0.9 to 3.2
Yes		Ref		Ref	
Number of iron tablets used					
<180	<0.0001	4.8	3.0 to 7.9	3.2	1.7 to 5.7
180 and more		Ref		Ref	
Weight gain					
Less than 6.53 kg	< 0.0001	3	1.9 to 4.8	2.6	1.5 to 4.7
6.53 kg and above		Ref		Ref	
Number of children					
1 (Primiparity)	0.03	1.9	1.2 to 3.1	1.8	0.9 to 3.6
3		0.9	0.4 to 2.2	0.4	0.1 to 1.2
4 and above		1.9	0.5 to 7.3	0.6	0.1 to 3.8
2		Ref		Ref	
Health problem (Co-morbidity)					
Yes	<0.0001	4.3	2.6 to 7.1	2.4	1.3 to 4.5
No		Ref		Ref	
Preterm birth					
Yes	<0.0001	3.9	2.2 to 7.2	2.9	1.4 to 6.1
No		Ref		Ref	

Table 2 shows the bivariate and multivariate analysis of dependent and independent variables. In bivariate analysis; support from husband during pregnancy, use of firewood and kerosene during cooking~~cooking material~~, having kitchen in the same living house~~location of kitchen~~, cigarette smoking by family members, cigarette smoking by mother during pregnancy, ~~food frequency during pregnancy,~~ use of additional food groups in their diet during pregnancy, four ANC visit as per protocol of Government of Nepal, ~~number of~~ iron tablets intake less than 180 tablets during pregnancy, weight gain during pregnancy less than 6.53 kg in-between second and third trimester, mother delivering her first baby~~number of children~~, health problem during pregnancy and preterm baby ~~are~~were associated with low birth weight. ~~of baby at 95% confidence interval.~~

In multivariate analysis, ~~location of the~~ having kitchen in the same living house (AOR 2.7, CI: 1.5-4.8), ~~number of~~ I~~i~~ron intake less than 180 tablets (AOR 3.2, CI: 1.7-5.7), maternal weight gain less than 6.53 kg during second and third trimester (AOR 2.6, CI: 1.5-4.7), ~~health~~

~~problem~~ co-morbidities during pregnancy (AOR 2.4, CI: 1.3- 4.5) and preterm birth (AOR 2.9, CI: 1.4-6.1) were significantly associated at 95% confidence interval with the low birth weight. ~~of the baby.~~ Similarly, age of mother, support from husband during pregnancy, ~~cooking material~~ use of firewood and kerosene during cooking, ~~the~~ smoking habit of the mother, smoking by family member, food frequency less than three per day, use of any additional food group ~~type of food used~~ during pregnancy, four ANC visit as per protocol, first~~number of~~ children was not associated with LBW in this study.

Discussion

This study ~~examined and~~ analyzed the socio-demographic factors, maternal factors and co-morbiditie~~sty~~ during recent pregnancy against low birth weight during delivery.

The maternal age is considered as a key factor for the healthy outcome of pregnancy. This study revealed no statistical association between maternal age and low birth weight which contradicts with the study done in Nepal, that shows a higher risk of delivering low birth weight babies by mother age less than 20 and more than 30 years (16, 17, 21). Smoking during pregnancy had a negative effect on the growth and development of the fetus because of chemical substances present in it. Nicotine present in the cigarette cause vasoconstriction resulting in the low oxygen flow to the fetus and Carbon-monoxide forms carboxyhemoglobin which inhibits the oxygen release to fetal tissues(22)~~.~~ In bivariate analysis mother habit of smoking had a higher risk of low birth weight in reference to the mother who did not smoke a cigarette (OR 6.3, 95% CI: 1.2-31.5). This finding is consistent with the findings of similar studies done in Bangladesh and Turkey (23, 24). Though there was a risk, however, there was no significant association between smoking and low birth

weight in multivariate analysis. This could be explained probably due to the small number of smokers in the study population. Moreover, it can also be explained by the social desirability bias, induced due to social stigma.

This study identified the location of the kitchen in the living house, iron intake less than 180 tablets, weight gain less than 6.53 kg during the second and third trimester, comorbidity during pregnancy, and preterm birth as the risk factors for low birth weight. The finding reveals that the cooking ~~stove~~ fuels namely firewood and kerosene use had a risk for LBW with reference to LPG and Biogas however, it was not ~~significantly~~ statistically significant. ~~different.~~ This finding contradicts to the find of the study done by Kadam YR et al. and Washam C (25, 26), however, this study revealed that ~~location of~~ having a kitchen in the same living house (proxy of indoor air pollution) had 2.5 times higher risk of delivering low birth weight which may be due to, living in the same house had higher risk and duration of exposure to the pollutants like $PM_{2.5}$, PM_{10}, NO_2, SO_2, CO ~~ion~~ caused by burning of fuels, ~~though the amount of exposure was not measured quantitatively,~~ leading to the impaired supply of oxygen, nutrition to the fetus resulting in the negative impact on the growth and development of fetus(27, 28). The amount of exposure was not measured quantitatively in this study.

This study showed that total iron tablet intake during pregnancy was associated with the birth weight of the child. Mothers who took less than 180 tablets of iron during their pregnancy were three times more likely to deliver low birth weight babies with reference to mothers who took iron equal to or more than 180 tablets during their pregnancy period (AOR 3.2, CI: 1.7-5.8). This finding is similar to the studies conducted in Nepal (16, 29). Low iron tablets intake causes the poor delivery of iron to the fetus thereby impair in proper hormonal and

neuronal regulation of pregnancy and poor oxygenation to the fetus leading to the poor growth and development of the fetus (30). However, the iron intake through diet during pregnancy was not measured in both cases and control.

The minimum standard weight gain during the second and third trimester is set as 6.53 kg (31). Women who gained weight less than 6.53 kg during the second and third trimester had 3 times higher risk of delivering low birth weight baby with reference to women whose weight gain was 6.53 kg or above (AOR 2.8, CI: 1.6-5.0). This finding is similar to the study done in Bangladesh (32) and Mozambique (33). The weight gain during pregnancy is impaired due to ill health, poor sanitation, and inadequate balance diet which at the end hamper the proper growth and development of the baby.

Women who had at least one health problem during their pregnancy were at higher risk of delivering low birth weight in comparison to women without any health problem (AOR 2.6, CI: 1.4-4.8). This finding is consistent with the study done in Nepal (17, 20). Likewise, this study suggest that mother delivering baby before completion of 37 weeks of gestation had higher risk of delivering low birth weight than the mothers who deliver the term baby (AOR 2.6, CI: 1.2-5.5) which is in line with the study done in Nepal (17), Ethiopia (34) and Kenya (35). Biologically it can be explained that preterm birth was less likely to get sufficient time for maturity, growth and nutrient intake which therefore can lead to low birth weight(36).

In this study, the researcher has retrieved maternal information namely gestational weight, iron tablets intake, gestational age, co-morbidity, frequency of ANC visits and birth weight of a baby from ANC card and maternity register to limit the recall bias. The selection of cases and controls were based on the records of maternal and neonatal register therefore, it is less likely that this study has misclassification biases both in the exposure and case-control

categories. Controls were selected randomly independent of the exposure status and moreover, as there was no non-responses rate in both the group, it is less likely that this study would suffer from selection biases.

Conclusion and recommendation

This study concluded that the ~~location~~ having~~of~~ the kitchen in the same living house (proxy of indoor air pollution), iron intake less than 180 tablets during pregnancy, weight gain less than 6.35 kg during the second and third trimester, co-morbidity during pregnancy, and preterm delivery were found to be associated risk factors of low birth weight. Thus, identified risk factors can be efficiently prevented through small doable actions that a family can apply and the mother can easily carry out. Maternal health programs can be directed towards motivating and tracking pregnant mothers for complete iron tablets intake during her pregnancy period. Intake of balance diet as per the protocol of the Government of Nepal for healthy growth and development of the child inside the uterus is of paramount importance. Family should help the mother for adequate rest, nutrition and healthy behavior to prevent risk factors identified in this study. ~~The identification of several risk factors of LBW in low resource settings can be dealt efficiently by engaging governmental and non-governmental agencies to prevent all the independent risk factors revealed in this study to promote the health and wellbeing of newborn babies.~~

Acknowledgment

Authors would like to thank Mr. Keshav Raj Pandit, Senior Public Health Administrator and the entire staff of DPHO, Dang for their kind support in the coordination with health

facilities, as well as to Female Community Health Volunteers, local leaders and teachers for their support during data collection. The authors would also like to express sincere thanks to all research participants for their valuable time and information.

Supporting information

All data files are available from the Figshare database

https://doi.org/10.6084/m9.figshare.11984433.v1

Formatted: Left

Formatted: Font: 18 pt, Bold, Do not check spelling or grammar

Reference

1. International Statistical Classification of Diseases and Related Health Problems- 10. Disorders related to short gestation and low birth weight, not elsewhere classified: World Health Organization; 2016.
2. Agbozo F, Abubakari A, Der J, Jahn A. Prevalence of low birth weight, macrosomia and stillbirth and their relationship to associated maternal risk factors in Hohoe Municipality, Ghana. Midwifery. 2016;40:200-6.
3. UNICEF, WHO. Low Birth Weight: Country, regional and global estimates. UNICEF, New York, 2004.
4. Mathewson KJ, Chow CH, Dobson KG, Pope EI, Schmidt LA, Van Lieshout RJ. Mental health of extremely low birth weight survivors: A systematic review and meta-analysis. Psychological bulletin. 2017;143(4):347-83.
5. Al Hazzani F, Al-Alaiyan S, Hassanein J, Khadawardi E. Short-term outcome of very low-birth-weight infants in a tertiary care hospital in Saudi Arabia. Annals of Saudi Medicine. 2011;31(6):581-5.
6. Chang H-Y, Sung Y-H, Wang S-M, Lung H-L, Chang J-H, Hsu C-H, et al. Short- and Long-Term Outcomes in Very Low Birth Weight Infants with Admission Hypothermia. PloS one. 2015;10(7):e0131976.
7. Fitzgibbons SC, Ching Y, Yu D, Carpenter J, Kenny M, Weldon C, et al. Mortality of necrotizing enterocolitis expressed by birth weight categories. Journal of Pediatric Surgery.44(6):1072-6.
8. Fan RG, Portuguez MW, Nunes ML. Cognition, behavior and social competence of preterm low birth weight children at school age. Clinics. 2013;68(7):915-21.
9. Sicuri E, Bardají A, Sigauque B, Maixenchs M, Nhacolo A, Nhalungo D, et al. Costs Associated with Low Birth Weight in a Rural Area of Southern Mozambique. PloS one. 2011;6(12):e28744.
10. Sharma D, Shastri S, Sharma P. Intrauterine Growth Restriction: Antenatal and Postnatal Aspects. Clinical medicine insights Pediatrics. 2016;10:67-83.

11. Cutland CL, Lackritz EM, Mallett-Moore T, Bardaji A, Chandrasekaran R, Lahariya C, et al. Low birth weight: Case definition & guidelines for data collection, analysis, and presentation of maternal immunization safety data. Vaccine. 2017;35(48 Pt A):6492-500.
12. Global Nutrition Targets 2025: Low Birth Weight Policy Brief. World Health Organization, 2014.
13. Ministry of Health Nepal, New ERA, and, ICF. Nepal Demographic and Health Survey 2016. Kathmandu, Nepal: Ministry of Health, New ERA, ICF International; 2017.
14. Malla M, Joshi DR, Chhetri K, Pandey P. Prevalence and contributing factors of low birth weight babies in institutional delvery. Journal of Patan Academy of Health Sciences. 2015;2(1):4.
15. Prajapati R, Shrestha S, Bhandari N. Prevalence and Associated Factors of Low Birth Weightamong Newborns in a Tertiary Level Hospital in Nepal. Kathmandu University Medical Journal. 2018;16(61):4.
16. Karuna Laxmi Shakya NS, Amod Kumar Poudyal, Rajendra Prasad Koju, Peter Oyloe and Sharad Raj Onta. Key factors associated with low birth weight at term in Nepal: A Case Control study. International Journal of Clinical and Biomedical Research. 2015;1(2):8.
17. Sharma SR, Giri S, Timalsina U, Bhandari SS, Basyal B, Wagle K, et al. Low birth weight at term and its determinants in a tertiary hospital of Nepal: a case-control study. PloS one. 2015;10(4):e0123962.
18. Yadav DK, Chaudhary U, Shrestha N. Risk factors associated with low birth weight. Journal of Nepal Health Research Council. 2011;9(2):159-64.
19. Kandel KP, Kafle S. Risk Factors Associated with Low Birth Weight among Deliveries at Bharatpur Hospital. Journal of Nepal Health Research Council. 2017;15(2):169-73.
20. Bhaskar RK, Deo KK, Neupane U, Chaudhary Bhaskar S, Yadav BK, Pokharel HP, et al. A Case Control Study on Risk Factors Associated with Low Birth Weight Babies in Eastern Nepal. International journal of pediatrics. 2015;2015:807373.
21. Yadav DK, Chaudhary U, Shrestha N. Risk Factors Associated with Low Birth Weight. Nepal Health Research Council. 2011;9(19):159-64.
22. Lambers DS, Clark KE. The maternal and fetal physiologic effects of nicotine. Seminars in Perinatology. 1996;20(2):115-26.
23. Hosain GM, Chatterjee N, Begum A, Saha SC. Factors associated with low birthweight in rural Bangladesh. Journal of tropical pediatrics. 2006;52(2):87-91.
24. Atessahin E, Pirincci E. Risk Factors Associated with Low Birth Weight Infants Born in Elazig, Eastern of Turkey. Iranian journal of public health. 2015;44(9):1299-300.
25. Kadam YR, Mimansa A, Chavan PV, Gore AD. Effect of prenatal exposure to kitchen fuel on birth weight. Indian journal of community medicine : official publication of Indian Association of Preventive & Social Medicine. 2013;38(4):212-6.
26. Washam C. Cooking with wood may fuel low birth weight: kitchen smoke puts babies at risk. Environmental health perspectives. 2008;116(4):A173-A.
27. Boy E, Bruce N, Delgado H. Birth weight and exposure to kitchen wood smoke during pregnancy in rural Guatemala. Environmental health perspectives. 2002;110(1):109-14.
28. Oliveira BFAd, Ignotti E, Hacon SS. A systematic review of the physical and chemical characteristics of pollutants from biomass burning and combustion of fossil fuels and health effects in Brazil. Cadernos de Saúde Pública. 2011;27:1678-98.

29. Bondevik GT, Lie RT, Ulstein M, Kvåle G. Maternal hematological status and risk of low birth weight and preterm delivery in Nepal. Acta Obstetricia et Gynecologica Scandinavica. 2001;80(5):402-8.
30. Beard JL. Micronutrient Deficiencies in the First Months of Life: Karger Publishers; 2004.
31. Coffey D. Prepregnancy body mass and weight gain during pregnancy in India and sub-Saharan Africa. Proceedings of the National Academy of Sciences. 2015;112(11):3302-7.
32. Hassoune S, Bassel S, Nani S, Elbouri H, Zine K, Maaroufi A. [Maternal factors associated with low birth weight: case-control study in a Moroccan public hospital]. The Pan African medical journal. 2015;20:303.
33. Osman N, Challis K, Cotiro M, Nordahl G, Berström S. Perinatal outcome in an obstetric cohort of Mozambican women. Journal of tropical pediatrics. 2001;47(1):30-8.
34. Gebremedhin M, Ambaw F, Admassu E, Berhane H. Maternal associated factors of low birth weight: a hospital based cross-sectional mixed study in Tigray, Northern Ethiopia. BMC pregnancy and childbirth. 2015;15:222.
35. Muchemi OM, Echoka E, Makokha A. Factors associated with low birth weight among neonates born at Olkalou District Hospital, Central Region, Kenya. The Pan African medical journal. 2015;20:108.
36. Negrato CA, Gomes MB. Low birth weight: causes and consequences. Diabetology & Metabolic Syndrome. 2013;5(1):49.

Dr. Pranil Man Singh Pradhan
Academic Editor
PLOS ONE

Dear Dr. Pradhan,

We thank you and the reviewers for a thorough reading and constructive feedback on our manuscript (PONE-D-19-31435) and for the opportunity to improve the original article. We are pleased to submit the revised research article titled, “Low birth weight and its associated risk factors: health facility-based case-control study” for your consideration in the PLOS ONE.

On behalf of my co-authors, we are sending the revised manuscript with track change and the revised manuscript. We believed that we had addressed all the comments from reviewers and hope that the revised manuscript is acceptable for publication in PLOS ONE.
On the following pages, you will find our response to the reviewer's comments. We thank you for your consideration of this resubmission.

We appreciate your time and look forward to your response.

Sincerely,
Anil K.C. (corresponding author)
kcanil707@gmail.com, +977-9847907142

Reviewer #1:

1. Introduction part needs more extensive literature review and rewrite your lines. eg-44-45

Revised: LBW is a valuable public health indicator of maternal health, nutrition, healthcare delivery, and poverty as LBW babies are at a higher risk of death and illness shortly after birth and non-communicable disease in the life course.

Added: The household cost, as well as health system costs, could be saved by reducing the burden of LBW.

Added: IUGR is the outcome of insufficient uterine–placental perfusion and fetal nutrition affecting the overall anthropometric parameter of the fetus. IUGR newborn has typical features of malnutrition. Extra-uterine infection, trauma, illness, IUGR, fetal infection, and anomalies are the contributing factors for preterm birth, resulting in growth retardation which ultimately results in LBW.

2. Identify the risk factors of LBW from literature, and see the factors associated in your context. If you are trying to establish the association in your local settings, then it should fulfill the various criteria, it cannot be just a random association

Yes, variables were selected based on studies done in Nepali and outside in a similar setting and consultation with experts and try to identify the possible risk factors in the local setting so, this is not just random association and possible explanation was done in the discussion section.

3. Methodology: Need more explanation on the tool you have used and its validity. Any specific reason to select only those variables in your study. Its not clear how you addressed your confounders

Revised: The tool was adapted from the previous study by Sharma SR et al. and other similar studies done in Nepal. The tool was translated into the Nepali language and pretested in Dhulikhel municipality; of Kavrepalanchok District among 10 percent of sample size i.e. 12 cases and 24 controls.

Response: Possible confounders were addressed by randomization and multivariate analysis.

4. Result: Some of your finding to do not meet the principle of Biological Plausibility

Response: The Biological plausibility of each finding from the multivariate analysis was explained in the discussion section.

Reviewer #2:

Note: Please find all the comments in the attached reviewed manuscript file in track change format.

Response: All the comments in the track change format are addressed individually.
Title of research: Clear

Abstract:

Organised well.

Line 28 and 29: Variables are not clear. This needs to be more specific like other variables. Suggestion: Not having a kitchen in the same living room.

Response: the variable location of kitchen wants to know about the kitchen in the same living house or separate from the living house. So it has been explained as having a kitchen in the same living house.

Line 33-35: In conclusion This seems recommendations. Please mention your conclusion based on your objective of this research only.

Revised: Having the kitchen in the same living house, iron intake less than 180 tablets during pregnancy, maternal weight gain less than 6.53 kg during the second and third trimester, co-morbidity during pregnancy and preterm birth were the risk factors associated with low birth weight.

Introduction:

I would suggest adding few literatures from Nepal as well.

Intext citation was added to highlight the literature that was reviewed from Nepal.

Methodology:

Line 54: I think this word (retrospective) is not required here.

Removed: An unmatched case-control study was used.

Line 56: Better to include health institutions.

Response: The data were taken from 31 health institutions of Dang district so it difficult to include the name of the health institution.

Revised: The study population was mothers, who delivered their babies in the governmental health institutions (28 birthing centers and 3 hospitals) of Dang from 17th August to 16th November 2018.

Line 67: Start from word…not from number.

Revised: One hundred and twenty-three

Line 68: Not clear….How did you select? Please be specific.

Added: One hundred and twenty-three cases were selected from the list of 224 cases and 246 controls from the list of 777 controls randomly independent of exposure status by generating random numbers The 123 cases and 246 controls with the highest random number were visited with the help of FCHV, local leaders, teachers for the data collection.

Results:

Line 88: Table 1: Rectify the included variables and better to concise the writing.

It means the kitchen is within the sleeping room in a house. What is the rationale of this category?

The variable wants to know whether the participants had a separate kitchen or not.

The rationale behind 6.53 Kg is, in our context it is difficult to know the exact weight gain during the pregnancy, however the weight gain in each ANC visit was recorded in both ANC card and maternity and newborn register. As per the study done in Low and middle-income county by Diane Coffey in 2015, concluded that the weight gain between the second and third trimester was 6.53Kg and this information can be easily accessible from ANC cards as well as maternity register.

Line 90-104: Add the clear component of variables

Added: Added as per comments.

Line 105: Table 2: Is the p-value same for both bivariate and multivariate analysis? (As you can see there is a change in Confidence Interval in bivariate and multivariate analysis.)

The P-value is of the chi-square test.

In the age of mothers, 20-30 years category as a reference is more scientific.

The reference category changed to 20-30 years and analysis did accordingly.

Other variable needs to be more specific. (Suggested in track change attached manuscript file.)

The location of the kitchen is the proxy indicator of Indoor air pollution.

Smoking by a family member is the proxy indicator of second-hand smoking.

Smoking habit indicates the habit of smoking any form of tobacco; Ciggrate, hukka and other traditional forms of smoking like rolling of tobacco in green leaf and smoke.

Food frequency per day: It is recommended that pregnant women should take one additional meal during their pregnancy for healthy growth and development of a fetus. Based on this line, we can assume that when she had taken an adequate diet or not, however, we cannot say it is balanced or not. It reveals the quantitative prospect of diet than qualitative.

Line 106: Here you said both bivariate and multivariate analysis shown in the table but in your title of the table only multivariate included. I think you need to format the table

The table has been formatted.

Discussion:

Line 126-129: You need to change your reference age group for statistical analysis. I already mentioned above in your result section.

Revised: The reference age group has been changed and statistical analysis was done accordingly.

Line 131: Better to add in-text citation

Added

Line 147; Mention supporting evidence with in-text citation.

Intext citation with supporting evidence has been added.

Line 166: Is this only clinical assessment?

It was based on the diagnosis made by health workers (any level) by reviewing cards.

Line 172: Better to mention in-text citation.

Mentioned

Line 178: How randomly? Need a better explanation.

Explained in the methodological section

Conclusion:

Line 191-193: But how?? You need more justification.

The recommendation was rearranged.

References:

Better to add doi and PMID numbers if available.

Added where possible.
