# Supplementary material for: Low birth weight and its associated risk factors: Health facility-based case-control study
Source: PLoS One. 2020 Jun 22;15(6):e0234907. doi: 10.1371/journal.pone.0234907 (PMC7307746; doi:10.1371/journal.pone.0234907)
Supplement: Attachment — Submitted filename: Manuscript_LBW_Reviewed ver 1-Dr Surya.doc [file pone.0234907.s003.doc]

Low birth weight and its associated risk factors: health facility-based case-control study

Anil K.C.^1*^, Prem Lal Basel^2^, Sarswoti Singh^3^

^1^ Health Foundation Nepal

^2^ Tribhuvan University Teaching Hospital, Department of Community Medicine and Public health

^3^ Tribhuvan University Teaching Hospital, Department of Community Medicine and Public health

* Corresponding author

E-mail: kcanil707@gmail.com

**Abstract**

**Background:** Low birth weight is a preventable public health problem. It is an important determinant of child survival and development, as well as long-term consequences like the onset of non-communicable disease in the life course. A large number of mortality and morbidity can be prevented by addressing the factors associated with low birth weight. The main objective of this study was to identify associated risk factors of low birth weight.

**Methodology:** A health facility-based unmatched case-control study was carried out from July 2018 to March 2019 among the mothers who delivered in health facilities of Dang district of Nepal from 17^th^ August to 16^th^ November 2018. The total sample size for the study was 369; 123 cases and 246 controls. Cases and controls were randomly selected independent of the exposure status in the ratio of 1:2. Information regarding exposure status was assessed through interviews and medical records. Mothers who delivered outside Dang districts were excluded from the study.

**Results:** Multivariate logistic regression found that the location of the kitchen in the same living house (AOR 2.7, CI: 1.5-4.8), iron intake less than 180 tablets (AOR 3.2, CI: 1.7-5.7), maternal weight gain during second and third trimester less than 6.53 kg (AOR 2.6, CI: 1.5-4.7), co-morbidity during pregnancy (AOR 2.4, CI: 1.3-4.5), preterm birth (AOR 2.9, CI: 1.4-6.1) were the risk factors associated with low birth weight.

**Conclusion:** The identified risk factors can be efficiently eliminated through behavior change communication programs focused on small doable actions that a family can apply and the mother can easily carry out.

**Introduction**

World Health Organization defines low birth weight (LBW) as the birth weight less than 2500 grams irrespective of gestational age . LBW is considered as a valuable public health indicator that reflects maternal health, nutrition, healthcare delivery. LBW babies are at a higher risk of death and illness shortly after birth and non-communicable disease in the life course . LBW infants are 20 times more likely to develop complications and die in compare to normal weight babies . LBW babies are in the potential risk of cognitive deficits, motor delays, cerebral palsy, and other behavior and psychological problem . The pathophysiology of low birth weight is unclear, whereas intrauterine growth retardation (IUGR) and preterm birth considered as the cause of LBW . LBW is considered a significant public health problem as it is estimated that 15% to 20% of all birth worldwide are LBW. The prevalence of LBW varies across regions with the highest 28% in South Asia and the lowest 6% in East Asia and the Pacific region . The prevalence of LBW in Nepal ranges from 12% to 21.6%, . A few descriptive and hospital based case-control studies have been done in Nepal. These studies could not represent the scenarios of risk factors of LBW at the community level. Hence this study aims to identify the scenario of associated risk factors of LBW at the community level.

**Methodology**

A retrospective unmatched case-control study was used. This study was conducted in Dang District of Nepal. The study population was mothers of children, who delivered their babies in health institutions of Dang from 17^th^ August to 16^th^ November 2018. The study population was divided into case and control as per the following definition.

**Case:** Mother delivering singleton live-born baby with birth weight less than 2500 grams without any congenital anomalies and were originally from Dang district.

**Control:** Mother delivering singleton live-born baby with birth weight more or equal to 2500 grams without any congenital anomalies and were from the same Dang district.

The sample size was calculated using EpiInfo software version 7. This was calculated by taking power at 80%, confident level as 95%, the percentage of control exposed as 65.40, the odds ratio of 2.06 from the maternal weight against LBW and the ratio of case to control was 1:2. The total sample size was 369 with 123 cases and 246 controls. The eligible numbers of research participants were enlisted from the maternal and neonate health register of 28 birthing centers and 3 hospitals of Dang District. 123 cases were selected from 224 cases and 246 controls from 777 controls randomly. Data were collected by scheduled interviews using semi-structured questionnaires, reviewing Antenatal Care (ANC) card and Maternal and newborn register.

Data entry was done in Epi data Version 3.1 following coding. Data analysis was done using SPSS software version 21. Bivariate associations between independent variables and low birth weight were tested through the Chi-square test and the association was analyzed by calculating crude odds ratios (OR) at 95% confidence interval through binary logistic regression. Multivariate logistic regression was examined for the relationship between independent variables and low birth weight to address the confounding effect. Hosmer and Lemeshow test was used to test the goodness-of-fit for regression models. The test statistic was 0.69 (p > 0.05) that showed that the model adequately fit the data.

Ethical clearance was obtained from the Institutional Review Committee (IRC) of the Institute of Medicine, Tribhuvan University. Permission was taken from the District Public Health Office (DPHO) Dang and respective health facilities. Written consent was taken from each participant after explaining the objectives of the study. After the interview, the mothers were informed about the importance of growth monitoring, exclusive breastfeeding, immunization and appropriate time of weaning.

**Results**

The mean age of the participants was 23 years (SD 4.4 years).

Table 1: Distribution of participants according to socio-demographic, maternal factors and co-morbidity during pregnancy

| Variables | Case (123) | Control (246) | Total (369) |
| --- | --- | --- | --- |
|  | n (%) | n (%) |  |
| Age of mother | | | |
| <20 Years | 35 (28.5%) | 55 (22.4%) | 90 (24.4%) |
| 20-30 Years | 79 (64.2%) | 178 (72.4%) | 257 (69.6%) |
| >30 years | 9 (7.3%) | 13 (5.3%) | 22 (6.0%) |
| Support from husband for what?? | | | |
| Yes | 102 (82.9%) | 222 (90.2%) | 324 (87.8%) |
| No | 21 (17.1%) | 24 (9.8%) | 45 (12.2%) |
| Cooking material | | | |
| Fire wood and Kerosene | 87 (70.7%) | 158 (64.2%) | 245 (66.4%) |
| LPG and Bio Gas | 36 (29.3%) | 88 (35.8%) | 124 (33.6%) |
| Location of kitchen | | | |
| Same house | 86 (69.9%) | 110 (44.7%) | 196 (53.1%) |
| Separate house and outside | 37 (30.1%) | 136 (55.3%) | 173 (46.9%) |
| Smoking by family member | | | |
| Yes | 65 (52.8%) | 100 (40.7%) | 165 (44.7%) |
| No | 58 (47.2%) | 146 (59.3%) | 204 (55.3%) |
| Smoking habit | | | |
| Yes | 6 (4.9%) | 2 (0.8%) | 8 (2.2%) |
| No | 117 (95.1%) | 244 (99.2%) | 361 (97.8%) |
| Food frequency per day | | | |
| Twice | 30 (24.4%) | 44 (17.9%) | 74 (20.1%) |
| Thrice | 81 (65.9%) | 166 (67.5%) | 247 (67%) |
| More | 12 (9.6%) | 36 (14.6%) | 48 (13.0%) |
| Type of food use | | | |
| As usual | 56 (45.5%) | 76 (30.9%) | 132 (35.8%) |
| Addition food (Any group) | 67 (54.5%) | 170 (69.1%) | 237 (64.2%) |
| ANC visit as per protocol | | | |
| Yes | 80 (65.0%) | 208 (84.6%) | 288 (78.0%) |
| No | 43 (35.0%) | 38 (15.4%) | 81 (22.0%) |
| Number of iron tablets used | | | |
| <180 | 60 (48.8%) | 40 (16.3%) | 100 (27.1%) |
| 180 and more | 63 (51.1%) | 206 (83.7%) | 269 (72.9%) |
| Weight gain between second and third trimester | | | |
| <6.53 kilogram (kg) | 91 (74.0%) | 120 (48.8%) | 211 (57.2%) |
| 6.53 kg and above | 32 (26.0%) | 126 (51.2%) | 158 (42.8%) |
| Number of children | | | |
| 1 | 79 (64.2%) | 121 (49.2%) | 200 (54.2%) |
| 2 | 32 (26.0%) | 93 (37.8%) | 125 (33.9%) |
| 3 | 8 (6.5%) | 26 (10.6%) | 34 (9.2%) |
| 4 and above | 4 (3.3%) | 6 (2.4%) | 10 (2.7%) |
| Health problem (Co morbidities) | | | |
| Yes | 50 (40.7%) | 34 (13.8%) | 84 (22.8%) |
| No | 73 (59.3%) | 212 (86.2%) | 285 (77.2%) |
| Preterm birth | | | |
| Yes | 33 (26.8%) | 21 (8.5%) | 54 (14.6%) |
| No | 90 (73.2%) | 225 (91.5%) | 315 (85.4%) |

Table 1 depicts thatgetting support from their husbands during pregnancy was common (87.8%). The major (66.4%)fuel used during cooking was firewood and keroseneThe majority (53.1%)of the household did not have a separate kitchen Majority (55.3%) of participants' family members did not smoke any form of cigarette. A small portion (…..??) of participants had the habit of smoking cigarettes during pregnancy. Two third (67%) of participants had their meal thrice a day and 64.2% had included additional food groups in their meal at the time of pregnancy. Majority (…??) of participants had attended ANC visit as per the protocol of the government of Nepal. Similarly, 73% of participants had taken 180 or more iron tablets during pregnancy. More than half of the participants had gained weight less than 6.53 kg during the second and third trimester. Fifty-four percentages of participants had one child. Majority (59.3% among cases and 86.2% among control) of the participants did not face any health problems (comorbidities) during their pregnancy and 14.6% of the babies were born before 37 weeks of gestation.

Table 2: Multivariate analysis of independent variables against low birth weight

| Variables | p-value) | Crude OR | CI (95%) | Adjusted OR | | CI (95%) |
| --- | --- | --- | --- | --- | --- | --- |
| Age of mother | | | | | | |
| <20 Years | 0.4 | 0.9 | 0.4 to 2.4 | 0.5 | | 0.1 to 2.2 |
| 20-30 Years |  | 0.6 | 0.3 to 1.6 | 0.7 | | 0.2 to 2.3 |
| >30 years |  | Ref |  | Ref | |  |
| Support from husband for what??? | | | | | | |
| No | 0.04 | 1.9 | 1.0 to 3.6 | 1.5 | | 0.7 to 3.3 |
| Yes |  | Ref |  | Ref | |  |
| Cooking material | | | | | | |
| Fire wood and Kerosene | 0.2 | 1.4 | 0.8 to 2.2 | 1.4 | | 0.7 to 2.6 |
| LPG and Bio Gas |  | Ref |  | Ref | |  |
| Location of kitchen | | | | | | |
| Same house | <0.0001 | 2.9 | 1.8 to 4.6 | 2.7 | | 1.5 to 4.8 |
| Separate house and outside |  | Ref |  | Ref | |  |
| Smoking by family member | | | | | | |
| Yes | 0.026 | 1.6 | 1.1 to 2.5 | 1.4 | | 0.8 to 2.5 |
| No |  | Ref |  | Ref | |  |
| Smoking habit | | | | | | |
| Yes | 0.032 | 6.3 | 1.2 to 31.5 | | 5.3 | 0.7 to 42.7 |
| No |  | Ref |  | | Ref |  |
| Food frequency per day | | | | | | |
| Twice | 0.19 | 2 | 0.9 to 4.6 | 1.3 | | 0.5 to 3.6 |
| Thrice |  | 1.5 | 0.7 to 3 | 1.5 | | 0.7 to 3.5 |
| More |  | Ref |  | Ref | |  |
| Type of food use | | | | | | |
| As usual | 0.006 | 1.9 | 1.2 to 2.9 | 0.9 | | 0.5 to 1.6 |
| Addition food (Any group) |  | Ref |  | Ref | |  |
| ANC visit as per protocol of Nepal government | | | | | | |
| No | <0.0001 | 2.9 | 1.8 to 4.9 | 1.7 | | 0.9 to 3.2 |
| Yes |  | Ref |  | Ref | |  |
| Number of iron tablets used | | | | | | |
| <180 | <0.0001 | 4.8 | 3.0 to 7.9 | 3.2 | | 1.7 to 5.7 |
| 180 and more |  | Ref |  | Ref | |  |
| Weight gain | | | | | | |
| Less than 6.53 kg | < 0.0001 | 3 | 1.9 to 4.8 | 2.6 | | 1.5 to 4.7 |
| 6.53 kg and above |  | Ref |  | Ref | |  |
| Number of children | | | | | | |
| 1 | 0.03 | 1.9 | 1.2 to 3.1 | 1.8 | | 0.9 to 3.6 |
| 3 |  | 0.9 | 0.4 to 2.2 | 0.4 | | 0.1 to 1.2 |
| 4 and above |  | 1.9 | 0.5 to 7.3 | 0.6 | | 0.1 to 3.8 |
| 2 |  | Ref |  | Ref | |  |
| Health problem (Associated co-morbidities) | | | | | | |
| Yes | <0.0001 | 4.3 | 2.6 to 7.1 | 2.4 | | 1.3 to 4.5 |
| No |  | Ref |  | Ref | |  |
| Preterm birth | | | | | | |
| Yes | <0.0001 | 3.9 | 2.2 to 7.2 | 2.9 | | 1.4 to 6.1 |
| No |  | Ref |  | Ref | |  |

Table 2 shows the bivariate and multivariate analysis of dependent and independent variables. In bivariate analysis; support from husband during pregnancy, what type…cooking material, where…location of kitchen, cigarette smoking by family members, cigarette smoking by mother during pregnancy, what type….food frequency during pregnancy, use of additional food groups in their diet during pregnancy, ANC visit as per protocol, how much…number of iron tablet intake, how much…weight gain during pregnancy, how many..number of children, health problem (comorbidities) during pregnancy and preterm baby are associated with low birth weight.

In multivariate analysis, where…location of the kitchen (AOR 2.7, CI: 1.5-4.8), how much…number of iron intake (AOR 3.2, CI: 1.7-5.7), how much…maternal weight gain during second and third trimester (AOR 2.6, CI: 1.5-4.7), health problem during pregnancy (AOR 2.4, CI: 1.3- 4.5) and preterm birth (AOR 2.9, CI: 1.4-6.1) were significantly associated at 95% confidence interval with the low birth weight. Similarly, age of mother, support from husband during pregnancy.., what type… cooking material, smoking habit of the mother, smoking by family member, how much..food frequency per day, which …type of food used, ANC visit as per protocol, how many…number of children was not associated with low birth weight.

**Discussion**

This study analysed the socio-demographic factors, maternal factors and co-morbiditiesduring recent pregnancy against low birth weight during delivery.

The maternal age is considered as a key factor for the healthy outcome of pregnancy. This study revealed no statistical association between maternal age and low birth weight which contradicts with the study done in Nepal, that shows a higher risk of delivering low birth weight babies by mother age less than 20 and more than 30 years . Smoking during pregnancy had a negative effect on the growth and development of the fetus because of chemical substances present in it. In bivariate analysis mother’s habit of smoking had a higher risk of low birth weight in reference to the mother who did not smoke a cigarette (OR 6.3, 95% CI: 1.2-31.5). This finding is consistent with the findings of similar studies done in Bangladesh and Turkey . Though there was a risk, however, there was no significant association between smoking and low birth weight in multivariate analysis. This could be explained probably due to the small number of smokers in the study population. Moreover, it can also be explained by the social desirability bias, induced due to social stigma.

This study identified the location of the kitchen in the living house, iron intake less than 180 tablets, weight gain less than 6.53 kg during the second and third trimester, comorbidity during pregnancy, and preterm birth as the risk factors for low birth weight. The finding reveals that the cooking fules namely firewood and kerosene use had a risk for LBW with reference to LPG and Biogas however, it was not statistically significant.. This finding contradicts to the find of the study done by Kadam YR et al. and Washam C , however this study revealed that location of kitchen in the same house had 2.5 times higher risk of delivering low birth weight which may be due to, living in the same house had higher risk and duration of exposure to the pollution caused by burning of fuels, though the amount of exposure was not measured quantitatively, leading to the negative impact on growth and development of fetus.

This study showed that total iron tablet intake during pregnancy was associated with the birth weight of the child. Mothers who took less than 180 tablets of iron during their pregnancy were three times more likely to deliver low birth weight babies with reference to mothers who took iron equal to or more than 180 tablets during their pregnancy period (AOR 3.2, CI: 1.7-5.8). This finding is similar to the studies conducted in Nepal . Low iron tablets intake causes the poor delivery of iron to the fetus thereby impair in proper hormonal and neuronal regulation of pregnancy and poor oxygenation to the fetus leading to the poor growth and development of the fetus . However, the iron intake through diet during pregnancy was not measured in both cases and control.

The minimum standard weight gain during the second and third trimester is set as 6.53 kg . Women who gained weight less than 6.53 kg during the second and third trimester had 3 times higher risk of delivering low birth weight baby with reference to women whose weight gain was 6.53 kg or above (AOR 2.8, CI: 1.6-5.0). This finding is similar to the study done in Bangladesh and Mozambique . The weight gain during pregnancy is impaired due to ill health, poor sanitation, and inadequate balance diet which at the end hamper the proper growth and development of the baby.

Women who had at least one health problem during their pregnancy were at higher risk of delivering low birth weight in comparison to women without any health problem (AOR 2.6, CI: 1.4-4.8). This finding is consistent with the study done in Nepal . Likewise this study suggest that mother delivering baby before completion of 37 weeks of gestation had higher risk of delivering low birth weight than the mothers who deliver the term baby (AOR 2.6, CI: 1.2-5.5) which is in line with the study done in Nepal , Ethiopia and Kenya . Biologically it can be explained that preterm birth was less likely to get sufficient time for maturity, growth and nutrient intake which therefore can lead to low birth weight.

In this study, the researcher has retrieved maternal information namely gestational weight, iron tablets intake, gestational age, co-morbidity, frequency of ANC visits and birth weight of a baby from ANC card and maternity register to limit the recall bias. The selection of cases and controls were based on the records of maternal and neonatal register therefore, it is less likely that this study has misclassification biases both in the exposure and case-control categories. Controls were selected randomly independent of the exposure status and moreover, as there was no non-responses rate in both the group, it is less likely that this study would suffer from selection biases.

**Conclusion and recommendation**

This study concluded that the location of the kitchen in the same living house (proxy of indoor air pollution), iron intake less than 180 tablets during pregnancy, weight gain less than 6.35 kg during the second and third trimester, co-morbidity during pregnancy, and preterm delivery were found to be associated risk factors of low birth weight. Thus, identified risk factors can be efficiently prevented through small doable actions that a family can apply and the mother can easily carry out. Maternal health programs can be directed towards motivating and tracking pregnant mothers for complete iron tablets intake during her pregnancy period. Intake of balance diet as per the protocol of the Government of Nepal for healthy growth and development of the child inside the uterus is of paramount importance. The identification of several risk factors of LBW in low resource settings can be dealt efficiently by engaging governmental and non-governmental agencies to prevent all the independent risk factors revealed in this study to promote the health and wellbeing of newborn babies.

**Acknowledgment**

Authors would like to thank Mr. Keshav Raj Pandit, Senior Public Health Administrator and the entire staff of DPHO, Dang for their kind support in the coordination with health facilities, as well as to Female Community Health Volunteers, local leaders and teachers for their support during data collection. The authors would also like to express sincere thanks to all research participants for their valuable time and information.

**Reference**

1.	International Statistical Classification of Diseases and Related Health Problems- 10. Disorders related to short gestation and low birth weight, not elsewhere classified: World Health Organization; 2016.

2.	Agbozo F, Abubakari A, Der J, Jahn A. Prevalence of low birth weight, macrosomia and stillbirth and their relationship to associated maternal risk factors in Hohoe Municipality, Ghana. Midwifery. 2016;40:200-6.

3.	UNICEF, WHO. Low Birth Weight: Country, regional and global estimates. UNICEF, New York, 2004.

4.	Mathewson KJ, Chow CH, Dobson KG, Pope EI, Schmidt LA, Van Lieshout RJ. Mental health of extremely low birth weight survivors: A systematic review and meta-analysis. Psychological bulletin. 2017;143(4):347-83.

5.	Al Hazzani F, Al-Alaiyan S, Hassanein J, Khadawardi E. Short-term outcome of very low-birth-weight infants in a tertiary care hospital in Saudi Arabia. Annals of Saudi Medicine. 2011;31(6):581-5.

6.	Chang H-Y, Sung Y-H, Wang S-M, Lung H-L, Chang J-H, Hsu C-H, et al. Short- and Long-Term Outcomes in Very Low Birth Weight Infants with Admission Hypothermia. PloS one. 2015;10(7):e0131976.

7.	Fitzgibbons SC, Ching Y, Yu D, Carpenter J, Kenny M, Weldon C, et al. Mortality of necrotizing enterocolitis expressed by birth weight categories. Journal of Pediatric Surgery.44(6):1072-6.

8.	Fan RG, Portuguez MW, Nunes ML. Cognition, behavior and social competence of preterm low birth weight children at school age. Clinics. 2013;68(7):915-21.

9.	Global Nutrition Targets 2025: Low Birth Weight Policy Brief. World Health Organization, 2014.

10.	Ministry of Health Nepal, New ERA, and, ICF. Nepal Demographic and Health Survey 2016. Kathmandu, Nepal: Ministry of Health, New ERA, ICF International; 2017.

11.	Malla M, Joshi DR, Chhetri K, Pandey P. Prevalence and contributing factors of low birth weight babies in institutional delvery. Journal of Patan Academy of Health Sciences. 2015;2(1):4.

12.	Prajapati R, Shrestha S, Bhandari N. Prevalence and Associated Factors of Low Birth Weightamong Newborns in a Tertiary Level Hospital in Nepal. Kathmandu University Medical Journal. 2018;16(61):4.

13.	Bhaskar RK, Deo KK, Neupane U, Chaudhary Bhaskar S, Yadav BK, Pokharel HP, et al. A Case Control Study on Risk Factors Associated with Low Birth Weight Babies in Eastern Nepal. International journal of pediatrics. 2015;2015:807373.

14.	Karuna Laxmi Shakya NS, Amod Kumar Poudyal, Rajendra Prasad Koju, Peter Oyloe and Sharad Raj Onta. Key factors associated with low birth weight at term in Nepal: A Case Control study. International Journal of Clinical and Biomedical Research. 2015;1(2):8.

15.	Yadav DK, Chaudhary U, Shrestha N. Risk Factors Associated with Low Birth Weight. Nepal Health Research Council. 2011;9(19):159-64.

16.	Sharma SR, Giri S, Timalsina U, Bhandari SS, Basyal B, Wagle K, et al. Low birth weight at term and its determinants in a tertiary hospital of Nepal: a case-control study. PloS one. 2015;10(4):e0123962.

17.	Hosain GM, Chatterjee N, Begum A, Saha SC. Factors associated with low birthweight in rural Bangladesh. Journal of tropical pediatrics. 2006;52(2):87-91.

18.	Atessahin E, Pirincci E. Risk Factors Associated with Low Birth Weight Infants Born in Elazig, Eastern of Turkey. Iranian journal of public health. 2015;44(9):1299-300.

19.	Kadam YR, Mimansa A, Chavan PV, Gore AD. Effect of prenatal exposure to kitchen fuel on birth weight. Indian journal of community medicine : official publication of Indian Association of Preventive & Social Medicine. 2013;38(4):212-6.

20.	Washam C. Cooking with wood may fuel low birth weight: kitchen smoke puts babies at risk. Environmental health perspectives. 2008;116(4):A173-A.

21.	Bondevik GT, Lie RT, Ulstein M, Kvåle G. Maternal hematological status and risk of low birth weight and preterm delivery in Nepal. Acta Obstetricia et Gynecologica Scandinavica. 2001;80(5):402-8.

22.	Beard JL. Micronutrient Deficiencies in the First Months of Life: Karger Publishers; 2004.

23.	Coffey D. Prepregnancy body mass and weight gain during pregnancy in India and sub-Saharan Africa. Proceedings of the National Academy of Sciences. 2015;112(11):3302-7.

24.	Hassoune S, Bassel S, Nani S, Elbouri H, Zine K, Maaroufi A. [Maternal factors associated with low birth weight: case-control study in a Moroccan public hospital]. The Pan African medical journal. 2015;20:303.

25.	Osman N, Challis K, Cotiro M, Nordahl G, Berström S. Perinatal outcome in an obstetric cohort of Mozambican women. Journal of tropical pediatrics. 2001;47(1):30-8.

26.	Gebremedhin M, Ambaw F, Admassu E, Berhane H. Maternal associated factors of low birth weight: a hospital based cross-sectional mixed study in Tigray, Northern Ethiopia. BMC pregnancy and childbirth. 2015;15:222.

27.	Muchemi OM, Echoka E, Makokha A. Factors associated with low birth weight among neonates born at Olkalou District Hospital, Central Region, Kenya. The Pan African medical journal. 2015;20:108.
