# Supplementary material for: Low birth weight and its associated risk factors: Health facility-based case-control study
Source: PLoS One. 2020 Jun 22;15(6):e0234907. doi: 10.1371/journal.pone.0234907 (PMC7307746; doi:10.1371/journal.pone.0234907)
Supplement: Attachment — Submitted filename: Response to Reviewers.docx [file pone.0234907.s005.docx]

Dr. Pranil Man Singh Pradhan

Academic Editor

PLOS ONE

Dear Dr. Pradhan,

We thank you and the reviewers for a thorough reading and constructive feedback on our manuscript (PONE-D-19-31435) and for the opportunity to improve the original article. We are pleased to submit the revised research article titled, “Low birth weight and its associated risk factors: health facility-based case-control study” for your consideration in the PLOS ONE.

On behalf of my co-authors, we are sending the revised manuscript with track change and the revised manuscript. We believed that we had addressed all the comments from reviewers and hope that the revised manuscript is acceptable for publication in PLOS ONE.

On the following pages, you will find our response to the reviewer's comments. We thank you for your consideration of this resubmission.

We appreciate your time and look forward to your response.

Sincerely,

Anil K.C. (corresponding author)

kcanil707@gmail.com, +977-9847907142

***Reviewer #1:***

*1. Introduction part needs more extensive literature review and rewrite your lines. eg-44-45*

Revised: LBW is a valuable public health indicator of maternal health, nutrition, healthcare delivery, and poverty as LBW babies are at a higher risk of death and illness shortly after birth and non-communicable disease in the life course.

Added: The household cost, as well as health system costs, could be saved by reducing the burden of LBW.

Added: IUGR is the outcome of insufficient uterine–placental perfusion and fetal nutrition affecting the overall anthropometric parameter of the fetus. IUGR newborn has typical features of malnutrition. Extra-uterine infection, trauma, illness, IUGR, fetal infection, and anomalies are the contributing factors for preterm birth, resulting in growth retardation which ultimately results in LBW.

*2. Identify the risk factors of LBW from literature, and see the factors associated in your context. If you are trying to establish the association in your local settings, then it should fulfill the various criteria, it cannot be just a random association*

Yes, variables were selected based on studies done in Nepali and outside in a similar setting and consultation with experts and try to identify the possible risk factors in the local setting so, this is not just random association and possible explanation was done in the discussion section.

*3. Methodology: Need more explanation on the tool you have used and its validity. Any specific reason to select only those variables in your study. Its not clear how you addressed your confounders*

Revised: The tool was adapted from the previous study by Sharma SR et al. and other similar studies done in Nepal. The tool was translated into the Nepali language and pretested in Dhulikhel municipality; of Kavrepalanchok District among 10 percent of sample size i.e. 12 cases and 24 controls.

Response: Possible confounders were addressed by randomization and multivariate analysis.

*4. Result: Some of your finding to do not meet the principle of Biological Plausibility*

Response: The Biological plausibility of each finding from the multivariate analysis was explained in the discussion section.

***Reviewer #2:***

*Note: Please find all the comments in the attached reviewed manuscript file in track change format.*

Response: All the comments in the track change format are addressed individually.
Title of research: Clear

Abstract:
Organised well.
*Line 28 and 29: Variables are not clear. This needs to be more specific like other variables. Suggestion: Not having a kitchen in the same living room.*

Response: the variable location of kitchen wants to know about the kitchen in the same living house or separate from the living house. So it has been explained as having a kitchen in the same living house.
*Line 33-35: In conclusion This seems recommendations. Please mention your conclusion based on your objective of this research only.*

Revised: Having the kitchen in the same living house, iron intake less than 180 tablets during pregnancy, maternal weight gain less than 6.53 kg during the second and third trimester, co-morbidity during pregnancy and preterm birth were the risk factors associated with low birth weight.

Introduction:
*I would suggest adding few literatures from Nepal as well.*

Intext citation was added to highlight the literature that was reviewed from Nepal.

Methodology:
*Line 54: I think this word (retrospective) is not required here.*

Removed: An unmatched case-control study was used.
*Line 56: Better to include health institutions.*

Response: The data were taken from 31 health institutions of Dang district so it difficult to include the name of the health institution.

Revised: The study population was mothers, who delivered their babies in the governmental health institutions (28 birthing centers and 3 hospitals) of Dang from 17th August to 16th November 2018.
*Line 67: Start from word…not from number.*

Revised: One hundred and twenty-three
*Line 68: Not clear….How did you select? Please be specific.*

Added: One hundred and twenty-three cases were selected from the list of 224 cases and 246 controls from the list of 777 controls randomly independent of exposure status by generating random numbers The 123 cases and 246 controls with the highest random number were visited with the help of FCHV, local leaders, teachers for the data collection.

Results:
*Line 88: Table 1: Rectify the included variables and better to concise the writing.
It means the kitchen is within the sleeping room in a house. What is the rationale of this category?*

The variable wants to know whether the participants had a separate kitchen or not.

The rationale behind 6.53 Kg is, in our context it is difficult to know the exact weight gain during the pregnancy, however the weight gain in each ANC visit was recorded in both ANC card and maternity and newborn register. As per the study done in Low and middle-income county by Diane Coffey in 2015, concluded that the weight gain between the second and third trimester was 6.53Kg and this information can be easily accessible from ANC cards as well as maternity register.

*Line 90-104: Add the clear component of variables*

Added: Added as per comments.  *Line 105: Table 2: Is the p-value same for both bivariate and multivariate analysis? (As you can see there is a change in Confidence Interval in bivariate and multivariate analysis.)*

The P-value is of the chi-square test. *In the age of mothers, 20-30 years category as a reference is more scientific.*

The reference category changed to 20-30 years and analysis did accordingly. *Other variable needs to be more specific. (Suggested in track change attached manuscript file.)*

The location of the kitchen is the proxy indicator of Indoor air pollution.

Smoking by a family member is the proxy indicator of second-hand smoking.

Smoking habit indicates the habit of smoking any form of tobacco; Ciggrate, hukka and other traditional forms of smoking like rolling of tobacco in green leaf and smoke.

Food frequency per day: It is recommended that pregnant women should take one additional meal during their pregnancy for healthy growth and development of a fetus. Based on this line, we can assume that when she had taken an adequate diet or not, however, we cannot say it is balanced or not. It reveals the quantitative prospect of diet than qualitative.

*Line 106: Here you said both bivariate and multivariate analysis shown in the table but in your title of the table only multivariate included. I think you need to format the table*

The table has been formatted.

**Discussion:**
*Line 126-129: You need to change your reference age group for statistical analysis. I already mentioned above in your result section.*

Revised: The reference age group has been changed and statistical analysis was done accordingly.

*Line 131: Better to add in-text citation*

Added

*Line 147; Mention supporting evidence with in-text citation.*

Intext citation with supporting evidence has been added.

*Line 166: Is this only clinical assessment?*

It was based on the diagnosis made by health workers (any level) by reviewing cards.

*Line 172: Better to mention in-text citation.*

Mentioned

*Line 178: How randomly? Need a better explanation.*

Explained in the methodological section

**Conclusion:** *Line 191-193: But how?? You need more justification.*

The recommendation was rearranged.

**References:** *Better to add doi and PMID numbers if available.*

Added where possible.
