# Supplementary material for: Low birth weight and its associated risk factors: Health facility-based case-control study
Source: PLoS One. 2020 Jun 22;15(6):e0234907. doi: 10.1371/journal.pone.0234907 (PMC7307746; doi:10.1371/journal.pone.0234907)
Supplement: Attachment — Submitted filename: Response to Reviewers.docx [file pone.0234907.s007.docx]

Dr. Pranil Man Singh Pradhan

Academic Editor

PLOS ONE

Dear Dr. Pradhan,

We thank you and the reviewers for a thorough reading and constructive feedback on our manuscript (PONE-D-19-31435) and for the opportunity to improve the original article. We are pleased to submit the revised research article titled, “Low birth weight and its associated risk factors: health facility-based case-control study” for your consideration in the PLOS ONE.

On behalf of my co-authors, we are sending the revised manuscript with track change and the revised manuscript. We believed that we had addressed all the comments from reviewers and hope that the revised manuscript is acceptable for publication in PLOS ONE.

On the following pages, you will find our response to the reviewer's comments. We thank you for your consideration of this resubmission.

We appreciate your time and look forward to your response.

Sincerely,

Anil K.C. (corresponding author)

kcanil707@gmail.com, +977-9847907142

***Reviewer #1:***

*1. Reviewer 1. The rationale for the study needs better justification as to how it better represents the community rather than hospital based studies done in the past (keeping in mind that the few of the study sites for this study were hospitals as well). Authors also need to highlight upon the limitations of the study. Authors need to revisit the grammatical errors and sentence structuring.*

Response: This study had taken research participants from 28 birthing centers and 3 hospitals so that it could better represent the community (Birthing center will represent the participants from rural areas and hospitals from both rural (referred case) and urban). So that it better represents the community. The limitation of the study was explained in the discussion section. Grammatical errors and sentence structuring were revisited and edited where necessary.

Added: However, the study had some limitations. The findings might be influenced by social desirability bias. The findings could not be generalized as the study was confined in the health institutions of one district. The details of comorbidity during pregnancy, the micro-nutritional status of the mother, and the quality of ANC visit were not evaluated which may affect the outcome of this study.

*Line 48-49: Please go through more literature and published articles on cause of LBW*

Response: The literature regarding the cause of LBW, which were reviewed were cited.

*Line 59: Do you want to present the scenario, identify the scenario of associated risk factors or provide the evidence of risk factors through its association?? Be clear on your research objectives and rationale.*

Response: The objective was to identify the risk factor of low birth weight. The sentence was rewritten as per the objective by removing the words “scenario of”.

*Line 63-68: Its better to put this paragraph below. First highlight the details of the methodology placed below this paragraph*

Response: Paragraph was placed as per comment below the detail of the methodology.

*Line 89: There should be good linkage between your study variables and questionnaire, please look into your conceptual framework, questionnaire and results, So that you could establish your association in your result section. select appropriate variables*

Response: The questionnaire was developed according to the conceptual framework and the result was generated based upon the data collected from the questionnaire. The whole steps were closely supervised by experts from the Institute of Medicine, Maharajgunj Medical Campus.

*Line 92: Please put this in Citation in the reference??*

Response: Updated as per comment.

*Line 108-109: You have kept this finding at first, is it that you find this result highly significant in your study???*

Response: It is not like that, I had mentioned the age of the mother above the table. I had re-arranged the findings in order of the variables on the table.

*Line 124-126: aren't these factors all related to smoke??? Biomass fuel/cigarette??*

Yes, these factors are related to smoke, however, the rate of exposure, chemical constituents, and the effects from them are different.

*Line 215: Spelling???*

Response: Spelling is changed
