# Supplementary material for: Low birth weight and its associated risk factors: Health facility-based case-control study
Source: PLoS One. 2020 Jun 22;15(6):e0234907. doi: 10.1371/journal.pone.0234907 (PMC7307746; doi:10.1371/journal.pone.0234907)
Supplement: Peer review history

# Peer review: Decision Letter 0

**DOI:** 10.1371/journal.pone.0234907.r001

**Authors:** Pranil Man Singh Pradhan

29 Jan 2020

PONE-D-19-31435

Low birth weight and its associated risk factors: health facility-based case-control study

PLOS ONE

Dear Mr. K C,

Thank you for submitting your manuscript to PLOS ONE. After careful consideration, we feel that it has merit but does not fully meet PLOS ONE’s publication criteria as it currently stands. Therefore, we invite you to submit a revised version of the manuscript that addresses the points raised during the review process.

We would appreciate receiving your revised manuscript by Mar 14 2020 11:59PM. When you are ready to submit your revision, log on to https://www.editorialmanager.com/pone/ and select the 'Submissions Needing Revision' folder to locate your manuscript file.

If you would like to make changes to your financial disclosure, please include your updated statement in your cover letter.

To enhance the reproducibility of your results, we recommend that if applicable you deposit your laboratory protocols in protocols.io, where a protocol can be assigned its own identifier (DOI) such that it can be cited independently in the future. For instructions see: http://journals.plos.org/plosone/s/submission-guidelines#loc-laboratory-protocols

Please include the following items when submitting your revised manuscript:

- A rebuttal letter that responds to each point raised by the academic editor and reviewer(s). This letter should be uploaded as separate file and labeled 'Response to Reviewers'.
- A marked-up copy of your manuscript that highlights changes made to the original version. This file should be uploaded as separate file and labeled 'Revised Manuscript with Track Changes'.
- An unmarked version of your revised paper without tracked changes. This file should be uploaded as separate file and labeled 'Manuscript'.

Please note while forming your response, if your article is accepted, you may have the opportunity to make the peer review history publicly available. The record will include editor decision letters (with reviews) and your responses to reviewer comments. If eligible, we will contact you to opt in or out.

We look forward to receiving your revised manuscript.

Kind regards,

Pranil Man Singh Pradhan

Academic Editor

PLOS ONE

Journal Requirements:

When submitting your revision, we need you to address these additional requirements:

1. Please ensure that your manuscript meets PLOS ONE's style requirements, including those for file naming. The PLOS ONE style templates can be found at http://www.plosone.org/attachments/PLOSOne_formatting_sample_main_body.pdf and http://www.plosone.org/attachments/PLOSOne_formatting_sample_title_authors_affiliations.pdf

2. Your ethics statement must appear in the Methods section of your manuscript. If your ethics statement is written in any section besides the Methods, please move it to the Methods section and delete it from any other section. Please also ensure that your ethics statement is included in your manuscript, as the ethics section of your online submission will not be published alongside your manuscript.

[Note: HTML markup is below. Please do not edit.]

Reviewers' comments:

Reviewer's Responses to Questions

**Comments to the Author**

1. Is the manuscript technically sound, and do the data support the conclusions?

The manuscript must describe a technically sound piece of scientific research with data that supports the conclusions. Experiments must have been conducted rigorously, with appropriate controls, replication, and sample sizes. The conclusions must be drawn appropriately based on the data presented. 

Reviewer #1: No

Reviewer #2: Partly

**********

2. Has the statistical analysis been performed appropriately and rigorously? 

Reviewer #1: Yes

Reviewer #2: No

**********

3. Have the authors made all data underlying the findings in their manuscript fully available?

The PLOS Data policy requires authors to make all data underlying the findings described in their manuscript fully available without restriction, with rare exception (please refer to the Data Availability Statement in the manuscript PDF file). The data should be provided as part of the manuscript or its supporting information, or deposited to a public repository. For example, in addition to summary statistics, the data points behind means, medians and variance measures should be available. If there are restrictions on publicly sharing data—e.g. participant privacy or use of data from a third party—those must be specified.

Reviewer #1: Yes

Reviewer #2: No

**********

4. Is the manuscript presented in an intelligible fashion and written in standard English?

PLOS ONE does not copyedit accepted manuscripts, so the language in submitted articles must be clear, correct, and unambiguous. Any typographical or grammatical errors should be corrected at revision, so please note any specific errors here.

Reviewer #1: No

Reviewer #2: Yes

**********

5. Review Comments to the Author

Please use the space provided to explain your answers to the questions above. You may also include additional comments for the author, including concerns about dual publication, research ethics, or publication ethics. (Please upload your review as an attachment if it exceeds 20,000 characters)

Reviewer #1: 1. Introduction part needs more extensive literature review and rewrite your lines. eg-44-45

2. Identify the risk factors of LBW from literature, and see the factors associated in your context. If you are trying to establish the association in your local settings, then it should fulfil the various criteria, it cannot be be just a random association.

3. You have tried to establish association in diverse area of risk factors, which is inconclusive. Needs more improvement in the technicality of hte subject matter

3. Methodology: Need more explanation on the tool you have used and its validity. Any specific reason to select only those variables in your study. Its not clear how you addressed your confounders.

4. Result: Some of your finding to do not meet the principle of Biological Plausibility

Reviewer #2: Note: Please find all the comments in the attached reviewed manuscript file in track change format.

Title of research: Clear

Abstract:

Organised well.

Line 28 and 29: Variables are not clear. This needs to be more specific like other variables. Suggestion: Not having a kitchen in the same living room.

Line 33-35: In conclusion_This seems recommendations. Please mention your conclusion based on your objective of this research only.

Introduction:

I would suggest adding few literatures from Nepal as well.

Methodology:

Line 54: I think this word (retrospective) is not required here.

Line 56: Better to include health institutions.

Line 67: Start from word…not from number.

Line 68: Not clear….How did you select? Please be specific.

Results:

Line 88: Table 1: Rectify the included variables and better to concise the writing.

It means the kitchen is within the sleeping room in a house. What is the rationale of this category?

Line 90-104: Add the clear component of variables

Line 105: Table 2: Is the p-value same for both bivariate and multivariate analysis? (As you can see there is a change in Confidence Interval in bivariate and multivariate analysis.)

In the age of mothers, 20-30 years category as a reference is more scientific.

Other variable needs to be more specific. (Suggested in track change attached manuscript file.)

Line 106: Here you said both bivariate and multivariate analysis shown in the table but in your title of the table only multivariate included. I think you need to format the table.

Discussion:

Line 126-129: You need to change your reference age group for statistical analysis. I already mentioned above in your result section.

Line 131: Better to add in text citation

Line 147; Mention supporting evidence with in text citation.

Line 166: Is this only clinical assessment?

Line 172: Better to mention in-text citation.

Line 178: How randomly? Need a better explanation.

Conclusion:

Line 191-193: But how?? You need more justification.

References:

Better to add doi and PMID numbers if available.

**********

6. PLOS authors have the option to publish the peer review history of their article (what does this mean?). If published, this will include your full peer review and any attached files.

If you choose “no”, your identity will remain anonymous but your review may still be made public.

**Do you want your identity to be public for this peer review?** For information about this choice, including consent withdrawal, please see our Privacy Policy.

Reviewer #1: No

Reviewer #2: Yes: Dr. Surya Bahadur Parajuli

[NOTE: If reviewer comments were submitted as an attachment file, they will be attached to this email and accessible via the submission site. Please log into your account, locate the manuscript record, and check for the action link "View Attachments". If this link does not appear, there are no attachment files to be viewed.]

While revising your submission, please upload your figure files to the Preflight Analysis and Conversion Engine (PACE) digital diagnostic tool, https://pacev2.apexcovantage.com/. PACE helps ensure that figures meet PLOS requirements. To use PACE, you must first register as a user. Registration is free. Then, login and navigate to the UPLOAD tab, where you will find detailed instructions on how to use the tool. If you encounter any issues or have any questions when using PACE, please email us at figures@plos.org. Please note that Supporting Information files do not need this step.

Attachment Submitted filename: PONE-D-19-31435_reviewewed_Dipendra Khatiwada.pdf Click here for additional data file.

Attachment Submitted filename: Manuscript_LBW_Reviewed ver 1-Dr Surya.doc Click here for additional data file.

Attachment Submitted filename: Manuscript_LBW_Reviewed ver 1-Dr Surya.pdf Click here for additional data file.

# Author comment: Author response to Decision Letter 0

**DOI:** 10.1371/journal.pone.0234907.r002

14 Mar 2020

Reviewer #1:

1. Introduction part needs more extensive literature review and rewrite your lines. eg-44-45

Revised: LBW is a valuable public health indicator of maternal health, nutrition, healthcare delivery, and poverty as LBW babies are at a higher risk of death and illness shortly after birth and non-communicable disease in the life course.

Added: The household cost, as well as health system costs, could be saved by reducing the burden of LBW.

Added: IUGR is the outcome of insufficient uterine–placental perfusion and fetal nutrition affecting the overall anthropometric parameter of the fetus. IUGR newborn has typical features of malnutrition. Extra-uterine infection, trauma, illness, IUGR, fetal infection, and anomalies are the contributing factors for preterm birth, resulting in growth retardation which ultimately results in LBW.

2. Identify the risk factors of LBW from literature, and see the factors associated in your context. If you are trying to establish the association in your local settings, then it should fulfill the various criteria, it cannot be just a random association

Yes, variables were selected based on studies done in Nepali and outside in a similar setting and consultation with experts and try to identify the possible risk factors in the local setting so, this is not just random association and possible explanation was done in the discussion section.

3. Methodology: Need more explanation on the tool you have used and its validity. Any specific reason to select only those variables in your study. Its not clear how you addressed your confounders

Revised: The tool was adapted from the previous study by Sharma SR et al. and other similar studies done in Nepal. The tool was translated into the Nepali language and pretested in Dhulikhel municipality; of Kavrepalanchok District among 10 percent of sample size i.e. 12 cases and 24 controls. 

Response: Possible confounders were addressed by randomization and multivariate analysis.

4. Result: Some of your finding to do not meet the principle of Biological Plausibility

Response: The Biological plausibility of each finding from the multivariate analysis was explained in the discussion section.

Reviewer #2: 

Note: Please find all the comments in the attached reviewed manuscript file in track change format.

Response: All the comments in the track change format are addressed individually.

Title of research: Clear

Abstract:

Organised well.

Line 28 and 29: Variables are not clear. This needs to be more specific like other variables. Suggestion: Not having a kitchen in the same living room.

Response: the variable location of kitchen wants to know about the kitchen in the same living house or separate from the living house. So it has been explained as having a kitchen in the same living house.

Line 33-35: In conclusion This seems recommendations. Please mention your conclusion based on your objective of this research only.

Revised: Having the kitchen in the same living house, iron intake less than 180 tablets during pregnancy, maternal weight gain less than 6.53 kg during the second and third trimester, co-morbidity during pregnancy and preterm birth were the risk factors associated with low birth weight.

Introduction:

I would suggest adding few literatures from Nepal as well.

Intext citation was added to highlight the literature that was reviewed from Nepal.

Methodology:

Line 54: I think this word (retrospective) is not required here.

Removed: An unmatched case-control study was used.

Line 56: Better to include health institutions.

Response: The data were taken from 31 health institutions of Dang district so it difficult to include the name of the health institution.

Revised: The study population was mothers, who delivered their babies in the governmental health institutions (28 birthing centers and 3 hospitals) of Dang from 17th August to 16th November 2018.

Line 67: Start from word…not from number.

Revised: One hundred and twenty-three

Line 68: Not clear….How did you select? Please be specific.

Added: One hundred and twenty-three cases were selected from the list of 224 cases and 246 controls from the list of 777 controls randomly independent of exposure status by generating random numbers The 123 cases and 246 controls with the highest random number were visited with the help of FCHV, local leaders, teachers for the data collection.

Results:

Line 88: Table 1: Rectify the included variables and better to concise the writing.

It means the kitchen is within the sleeping room in a house. What is the rationale of this category?

The variable wants to know whether the participants had a separate kitchen or not. 

The rationale behind 6.53 Kg is, in our context it is difficult to know the exact weight gain during the pregnancy, however the weight gain in each ANC visit was recorded in both ANC card and maternity and newborn register. As per the study done in Low and middle-income county by Diane Coffey in 2015, concluded that the weight gain between the second and third trimester was 6.53Kg and this information can be easily accessible from ANC cards as well as maternity register.

Line 90-104: Add the clear component of variables

Added: Added as per comments. 

Line 105: Table 2: Is the p-value same for both bivariate and multivariate analysis? (As you can see there is a change in Confidence Interval in bivariate and multivariate analysis.)

The P-value is of the chi-square test.

In the age of mothers, 20-30 years category as a reference is more scientific.

The reference category changed to 20-30 years and analysis did accordingly.

Other variable needs to be more specific. (Suggested in track change attached manuscript file.)

The location of the kitchen is the proxy indicator of Indoor air pollution.

Smoking by a family member is the proxy indicator of second-hand smoking.

Smoking habit indicates the habit of smoking any form of tobacco; Ciggrate, hukka and other traditional forms of smoking like rolling of tobacco in green leaf and smoke.

Food frequency per day: It is recommended that pregnant women should take one additional meal during their pregnancy for healthy growth and development of a fetus. Based on this line, we can assume that when she had taken an adequate diet or not, however, we cannot say it is balanced or not. It reveals the quantitative prospect of diet than qualitative. 

Line 106: Here you said both bivariate and multivariate analysis shown in the table but in your title of the table only multivariate included. I think you need to format the table

The table has been formatted.

Discussion:

Line 126-129: You need to change your reference age group for statistical analysis. I already mentioned above in your result section.

Revised: The reference age group has been changed and statistical analysis was done accordingly.

Line 131: Better to add in-text citation

Added

Line 147; Mention supporting evidence with in-text citation.

Intext citation with supporting evidence has been added.

Line 166: Is this only clinical assessment?

It was based on the diagnosis made by health workers (any level) by reviewing cards.

Line 172: Better to mention in-text citation.

Mentioned

Line 178: How randomly? Need a better explanation.

Explained in the methodological section

Conclusion:

Line 191-193: But how?? You need more justification.

The recommendation was rearranged.

References:

Better to add doi and PMID numbers if available.

Added where possible.

Attachment Submitted filename: Response to Reviewers.docx Click here for additional data file.

# Peer review: Decision Letter 1

**DOI:** 10.1371/journal.pone.0234907.r003

**Authors:** Pranil Man Singh Pradhan

28 Apr 2020

PONE-D-19-31435R1

Low birth weight and its associated risk factors: health facility-based case-control study

PLOS ONE

Dear Mr. K C,

Thank you for submitting your manuscript to PLOS ONE. After careful consideration, we feel that it has merit but does not fully meet PLOS ONE’s publication criteria as it currently stands. Therefore, we invite you to submit a revised version of the manuscript that addresses the points raised during the review process.

We would appreciate receiving your revised manuscript by Jun 12 2020 11:59PM. When you are ready to submit your revision, log on to https://www.editorialmanager.com/pone/ and select the 'Submissions Needing Revision' folder to locate your manuscript file.

If you would like to make changes to your financial disclosure, please include your updated statement in your cover letter.

To enhance the reproducibility of your results, we recommend that if applicable you deposit your laboratory protocols in protocols.io, where a protocol can be assigned its own identifier (DOI) such that it can be cited independently in the future. For instructions see: http://journals.plos.org/plosone/s/submission-guidelines#loc-laboratory-protocols

Please include the following items when submitting your revised manuscript:

- A rebuttal letter that responds to each point raised by the academic editor and reviewer(s). This letter should be uploaded as separate file and labeled 'Response to Reviewers'.
- A marked-up copy of your manuscript that highlights changes made to the original version. This file should be uploaded as separate file and labeled 'Revised Manuscript with Track Changes'.
- An unmarked version of your revised paper without tracked changes. This file should be uploaded as separate file and labeled 'Manuscript'.

Please note while forming your response, if your article is accepted, you may have the opportunity to make the peer review history publicly available. The record will include editor decision letters (with reviews) and your responses to reviewer comments. If eligible, we will contact you to opt in or out.

We look forward to receiving your revised manuscript.

Kind regards,

Pranil Man Singh Pradhan

Academic Editor

PLOS ONE

Additional Editor Comments (if provided):

Kindly address the comments provided by Reviewer 1. The rationale for the study needs better justification as to how it better represents the community rather than hospital based studies done in the past (keeping in mind that the few of the study sites for this study were hospitals as well). Authors also need to highlight upon the limitations of the study. Authors need to revisit the grammatical errors and sentence structuring.

[Note: HTML markup is below. Please do not edit.]

Reviewers' comments:

Reviewer's Responses to Questions

**Comments to the Author**

1. If the authors have adequately addressed your comments raised in a previous round of review and you feel that this manuscript is now acceptable for publication, you may indicate that here to bypass the “Comments to the Author” section, enter your conflict of interest statement in the “Confidential to Editor” section, and submit your "Accept" recommendation.

Reviewer #1: (No Response)

Reviewer #2: All comments have been addressed

**********

2. Is the manuscript technically sound, and do the data support the conclusions?

The manuscript must describe a technically sound piece of scientific research with data that supports the conclusions. Experiments must have been conducted rigorously, with appropriate controls, replication, and sample sizes. The conclusions must be drawn appropriately based on the data presented. 

Reviewer #1: No

Reviewer #2: Yes

**********

3. Has the statistical analysis been performed appropriately and rigorously? 

Reviewer #1: Yes

Reviewer #2: Yes

**********

4. Have the authors made all data underlying the findings in their manuscript fully available?

The PLOS Data policy requires authors to make all data underlying the findings described in their manuscript fully available without restriction, with rare exception (please refer to the Data Availability Statement in the manuscript PDF file). The data should be provided as part of the manuscript or its supporting information, or deposited to a public repository. For example, in addition to summary statistics, the data points behind means, medians and variance measures should be available. If there are restrictions on publicly sharing data—e.g. participant privacy or use of data from a third party—those must be specified.

Reviewer #1: Yes

Reviewer #2: Yes

**********

5. Is the manuscript presented in an intelligible fashion and written in standard English?

PLOS ONE does not copyedit accepted manuscripts, so the language in submitted articles must be clear, correct, and unambiguous. Any typographical or grammatical errors should be corrected at revision, so please note any specific errors here.

Reviewer #1: No

Reviewer #2: Yes

**********

6. Review Comments to the Author

Please use the space provided to explain your answers to the questions above. You may also include additional comments for the author, including concerns about dual publication, research ethics, or publication ethics. (Please upload your review as an attachment if it exceeds 20,000 characters)

Reviewer #1: Please see the comments in the manuscript itself. Please look into the selection of variables and draw appropriate variable from your conceptual framework to develop your variables.

Reviewer #2: The authors addressed all my comments and feedback. We can proceed for publication of this research article.

**********

7. PLOS authors have the option to publish the peer review history of their article (what does this mean?). If published, this will include your full peer review and any attached files.

If you choose “no”, your identity will remain anonymous but your review may still be made public.

**Do you want your identity to be public for this peer review?** For information about this choice, including consent withdrawal, please see our Privacy Policy.

Reviewer #1: No

Reviewer #2: Yes: Dr. Surya B. Parajuli

[NOTE: If reviewer comments were submitted as an attachment file, they will be attached to this email and accessible via the submission site. Please log into your account, locate the manuscript record, and check for the action link "View Attachments". If this link does not appear, there are no attachment files to be viewed.]

While revising your submission, please upload your figure files to the Preflight Analysis and Conversion Engine (PACE) digital diagnostic tool, https://pacev2.apexcovantage.com/. PACE helps ensure that figures meet PLOS requirements. To use PACE, you must first register as a user. Registration is free. Then, login and navigate to the UPLOAD tab, where you will find detailed instructions on how to use the tool. If you encounter any issues or have any questions when using PACE, please email us at figures@plos.org. Please note that Supporting Information files do not need this step.

Attachment Submitted filename: PONE-D-19-31435_R1_reviewer.pdf Click here for additional data file.

# Author comment: Author response to Decision Letter 1

**DOI:** 10.1371/journal.pone.0234907.r004

1 Jun 2020

Reviewer #1:

1. Reviewer 1. The rationale for the study needs better justification as to how it better represents the community rather than hospital based studies done in the past (keeping in mind that the few of the study sites for this study were hospitals as well). Authors also need to highlight upon the limitations of the study. Authors need to revisit the grammatical errors and sentence structuring.

Response: This study had taken research participants from 28 birthing centers and 3 hospitals so that it could better represent the community (Birthing center will represent the participants from rural areas and hospitals from both rural (referred case) and urban). So that it better represents the community. The limitation of the study was explained in the discussion section. Grammatical errors and sentence structuring were revisited and edited where necessary.

Added: However, the study had some limitations. The findings might be influenced by social desirability bias. The findings could not be generalized as the study was confined in the health institutions of one district. The details of comorbidity during pregnancy, the micro-nutritional status of the mother, and the quality of ANC visit were not evaluated which may affect the outcome of this study.

Line 48-49: Please go through more literature and published articles on cause of LBW

Response: The literature regarding the cause of LBW, which were reviewed were cited.

Line 59: Do you want to present the scenario, identify the scenario of associated risk factors or provide the evidence of risk factors through its association?? Be clear on your research objectives and rationale.

Response: The objective was to identify the risk factor of low birth weight. The sentence was rewritten as per the objective by removing the words “scenario of”.

Line 63-68: Its better to put this paragraph below. First highlight the details of the methodology placed below this paragraph

Response: Paragraph was placed as per comment below the detail of the methodology.

Line 89: There should be good linkage between your study variables and questionnaire, please look into your conceptual framework, questionnaire and results, So that you could establish your association in your result section. select appropriate variables

Response: The questionnaire was developed according to the conceptual framework and the result was generated based upon the data collected from the questionnaire. The whole steps were closely supervised by experts from the Institute of Medicine, Maharajgunj Medical Campus.

Line 92: Please put this in Citation in the reference??

Response: Updated as per comment.

Line 108-109: You have kept this finding at first, is it that you find this result highly significant in your study???

Response: It is not like that, I had mentioned the age of the mother above the table. I had re-arranged the findings in order of the variables on the table.

Line 124-126: aren't these factors all related to smoke??? Biomass fuel/cigarette??

Yes, these factors are related to smoke, however, the rate of exposure, chemical constituents, and the effects from them are different.

Line 215: Spelling???

Response: Spelling is changed

Attachment Submitted filename: Response to Reviewers.docx Click here for additional data file.

# Editor report: Decision Letter 2

**DOI:** 10.1371/journal.pone.0234907.r005

**Authors:** Pranil Man Singh Pradhan

5 Jun 2020

Low birth weight and its associated risk factors: health facility-based case-control study

PONE-D-19-31435R2

Dear Dr. K C,

We’re pleased to inform you that your manuscript has been judged scientifically suitable for publication and will be formally accepted for publication once it meets all outstanding technical requirements.

Within one week, you’ll receive an e-mail detailing the required amendments. When these have been addressed, you’ll receive a formal acceptance letter and your manuscript will be scheduled for publication.

An invoice for payment will follow shortly after the formal acceptance. To ensure an efficient process, please log into Editorial Manager at http://www.editorialmanager.com/pone/, click the 'Update My Information' link at the top of the page, and double check that your user information is up-to-date. If you have any billing related questions, please contact our Author Billing department directly at authorbilling@plos.org.

If your institution or institutions have a press office, please notify them about your upcoming paper to help maximize its impact. If they’ll be preparing press materials, please inform our press team as soon as possible -- no later than 48 hours after receiving the formal acceptance. Your manuscript will remain under strict press embargo until 2 pm Eastern Time on the date of publication. For more information, please contact onepress@plos.org.

Kind regards,

Pranil Man Singh Pradhan

Academic Editor

PLOS ONE

Additional Editor Comments (optional):

Reviewers' comments:

# Editor report: Acceptance letter

**DOI:** 10.1371/journal.pone.0234907.r006

**Authors:** Pranil Man Singh Pradhan

10 Jun 2020

PONE-D-19-31435R2 

Low birth weight and its associated risk factors: health facility-based case-control study 

Dear Dr. K C:

I'm pleased to inform you that your manuscript has been deemed suitable for publication in PLOS ONE. Congratulations! Your manuscript is now with our production department. 

If your institution or institutions have a press office, please let them know about your upcoming paper now to help maximize its impact. If they'll be preparing press materials, please inform our press team within the next 48 hours. Your manuscript will remain under strict press embargo until 2 pm Eastern Time on the date of publication. For more information please contact onepress@plos.org.

If we can help with anything else, please email us at plosone@plos.org. 

Thank you for submitting your work to PLOS ONE and supporting open access. 

Kind regards, 

PLOS ONE Editorial Office Staff

on behalf of

Dr. Pranil Man Singh Pradhan 

Academic Editor

PLOS ONE
